# Copy number variation of *TdDof* controls solid-stemmed architecture in wheat

**DOI:** 10.1073/pnas.2009418117

**Published:** 2020-10-30

**Authors:** Kirby T. Nilsen, Sean Walkowiak, Daoquan Xiang, Peng Gao, Teagen D. Quilichini, Ian R. Willick, Brook Byrns, Amidou N’Diaye, Jennifer Ens, Krystalee Wiebe, Yuefeng Ruan, Richard D. Cuthbert, Melanie Craze, Emma J. Wallington, James Simmonds, Cristobal Uauy, Raju Datla, Curtis J. Pozniak

**Affiliations:** ^a^Crop Development Centre and Department of Plant Sciences, College of Agriculture and Bioresources, University of Saskatchewan, Saskatoon, SK S7N 5A8, Canada;; ^b^Brandon Research and Development Centre, Agriculture and Agri-Food Canada, Brandon, MB R7A 5Y3, Canada;; ^c^Grain Research Laboratory, Canadian Grain Commission, Winnipeg, MB R3C 3G8, Canada;; ^d^Aquatic and Crop Resource Development Research Centre, National Research Council Canada, Saskatoon, SK S7N 0W9, Canada;; ^e^Global Institute for Food Security, University of Saskatchewan, Saskatoon, SK S7N 4J8, Canada;; ^f^Swift Current Research and Development Centre, Agriculture and Agri-Food Canada, Swift Current, SK S9H 3X2, Canada;; ^g^NIAB, Cambridge CB3 OLE, United Kingdom;; ^h^John Innes Centre, Norwich NR4 7UH, United Kingdom

**Keywords:** solid stem, wheat, copy number variation (CNV), Dof transcription factor, programmed cell death (PCD)

## Abstract

Stem solidness is an important agronomic trait of durum (*Triticum turgidum* L. var. *durum*) and bread (*Triticum aestivum* L.) wheat that provides resistance to the wheat stem sawfly. This dominant trait is conferred by the *SSt1* locus on chromosome 3B. However, the molecular identity and mechanisms underpinning stem solidness have not been identified. Here, we demonstrate that copy number variation of *TdDof*, a gene encoding a putative DNA binding with one finger protein, controls the stem solidness trait in wheat. Using map-based cloning, we localized *TdDof* to within a physical interval of 2.1 Mb inside the *SSt1* locus. Molecular analysis revealed that hollow-stemmed wheat cultivars such as Kronos carry a single copy of *TdDof*, whereas solid-stemmed cultivars such as CDC Fortitude carry multiple identical copies of the gene. Deletion of all *TdDof* copies from CDC Fortitude resulted in the loss of stem solidness, whereas the transgenic overexpression of *TdDof* restored stem solidness in the *TdDof* deletion mutant *pithless1 *and conferred stem solidness in Kronos. In solid-stemmed cultivars, increased *TdDof* expression was correlated with the down-regulation of genes whose orthologs have been implicated in programmed cell death (PCD) in other species. Anatomical and histochemical analyses revealed that hollow-stemmed lines had stronger PCD-associated signals in the pith cells compared to solid-stemmed lines, which suggests copy number-dependent expression of *TdDof* could be directly or indirectly involved in the negative regulation of PCD. These findings provide opportunities to manipulate stem development in wheat and other monocots for agricultural or industrial purposes.

## Significance

Solid-stemmed wheat cultivars are resistant to the wheat stem sawfly, an important agricultural pest. Here, we identify *TdDof* as the causal gene that controls stem solidness in wheat. We show that copy number gain of *TdDof* correlates with its increased expression and the solid-stem phenotype. Our results suggest *TdDof* could function as a key regulator of genes involved in programmed cell death of the pith parenchyma cells. This research provides the framework to manipulate stem architecture in wheat and other monocots, which can be applied toward downstream agricultural and industrial applications. These include enhancing wheat stem sawfly resistance, modifying carbon partitioning and water-soluble carbohydrate remobilization in plants under drought and temperature stress, and bioenergy production.

Wheat is the world’s most widely grown crop, with annual global production of ∼750 million metric tons (1). The two most commonly grown types are bread wheat (*Triticum aestivum* L.) used for bread making and biscuits, and durum wheat (*Triticum turgidum* L. var. *durum*) used to produce pasta and couscous. Wheat consumption accounts for ∼20% of the protein and calories consumed by humans (1). By 2050, the demand for wheat is predicted to increase by 50% to sustain a global population projected to surpass 9 billion people (1). Wheat production is threatened by the changing climate, which is expected to produce more severe and prolonged periods of extreme weather events such as heat, drought, and cold. Continuously evolving pathogen and insect populations also threaten wheat production on a global scale. A better understanding of the key genes involved in the physiological traits of wheat, including genes controlling stem development, will help breeders develop resilient cultivars equipped for these challenges to secure sustainable global food production.

The wheat stem supports the leaves and grain-yielding inflorescence (spike), transporting water and minerals from the soil to the aboveground portions of the plant via the interconnected cells that form the xylem (2). Photoassimilates from source tissues are translocated through the stem phloem to sink tissues. Water-soluble carbohydrates (WSC), and, to a lesser degree, starch, are stored in the stem and are later remobilized during grain filling (3). Fructan, the primary carbon storage molecule in the wheat stem, accumulates during periods of slow growth or when photosynthetic rates exceed sink tissue demands (4). The remobilization of water and WSC from the stem to the developing grain not only increases grain yield but also provides protection under moisture deficit (5, 6).

The wheat stem is divided into nodes to which the leaf sheaths are attached, and the regions between nodes are known as internodes. Between four and seven internodes elongate from a single internode primordium, depending on genetic and environmental factors (2). Stem elongation is a developmentally regulated process that begins from one of the lowermost nodes and progresses in succession upward toward the peduncle, which elongates last to support the spike. The intercalary meristem near the base of each internode gives rise to ground tissue comprising parenchyma cells, which differentiate to form the pith, and vascular strands that subsequently differentiate into vascular tissues surrounded by the cortex. In mature wheat stems, vascular bundles are separated by a layer of interfascicular parenchyma cells arranged in a ring surrounding a central cavity (internodal lacuna), which is formed by the collapse and breakdown of parenchyma cells that form the pith and causes the stems to become hollow (2). Pith is composed of undifferentiated parenchyma cells, which play important roles in water and WSC (i.e., sucrose, glucose, fructose, and fructan) storage (7). In moisture-limiting environments, the water-holding capacity of the pith parenchyma is proposed to be an important driver of drought and heat tolerance (8).

In some wheat cultivars, stem development has been reprogrammed to avoid the formation of a hollow stem. The main genetic factor conferring stem solidness resides within a major quantitative trait locus on chromosome 3B in durum (*SSt1*) and bread wheat (*Qss.msub-3BL*), hereafter referred to as *SSt1* (9–11). The underlying gene, however, has not been identified. Several sources have been used to introduce stem solidness into elite breeding lines, including the commonly used Portuguese wheat landrace S-615 for bread wheat (12), and the German cultivar Biodur for durum wheat (13, 14). The trait in durum wheat is thought to have originated in the North African landrace Golden Ball. In all of these cultivars, the culm becomes partially or completely filled with pith (7).

The wheat stem sawfly (WSS) *Cephus cinctus* Norton (Hymenoptera: Cephidae) is a major pest in nearly the entire durum wheat-growing region of North America. Initially considered to be a pest of wild grasses, WSS developed a strong preference for wheat during the rapid expansion of this crop across the North American prairies beginning in the late 19th century (15). The ability of WSS to adapt to host preferences and thrive in new agroecological environments poses a risk to the geographical expansion of wheat that is expected to occur due to climate change. As sawfly populations also exist in Asia and Europe, they could pose a significant threat to wheat production if a similar shift in host preference were to occur (16). In North America, harvest losses attributed to WSS are estimated to exceed $350 million per year (15). Over the last century, the most effective way to minimize damage caused by WSS has been to grow solid-stemmed wheat cultivars (17). Solid stems provide resistance to WSS by deterring oviposition, and impeding larval development and growth inside the stem (18).

In the current study, we generate molecular and functional evidence that *TdDof* is the causal gene modulating stem solidness in wheat. Solid-stemmed cultivars carry multiple copies of *TdDof*, which correspond to its higher expression levels and the solid-stemmed phenotype. Our findings provide insight into putative *TdDof-*mediated regulatory functions associated with solid-stemmed architecture in wheat that may include regulation of key genes involved in programmed cell death (PCD). In addition, our results lay the foundation for developing genetic markers for screening and selecting desirable alleles for breeding wheat cultivars with the stem solidness trait.

## Results

### Fine Mapping of the *SSt1* Genomic Interval.

**Fig. 1.**
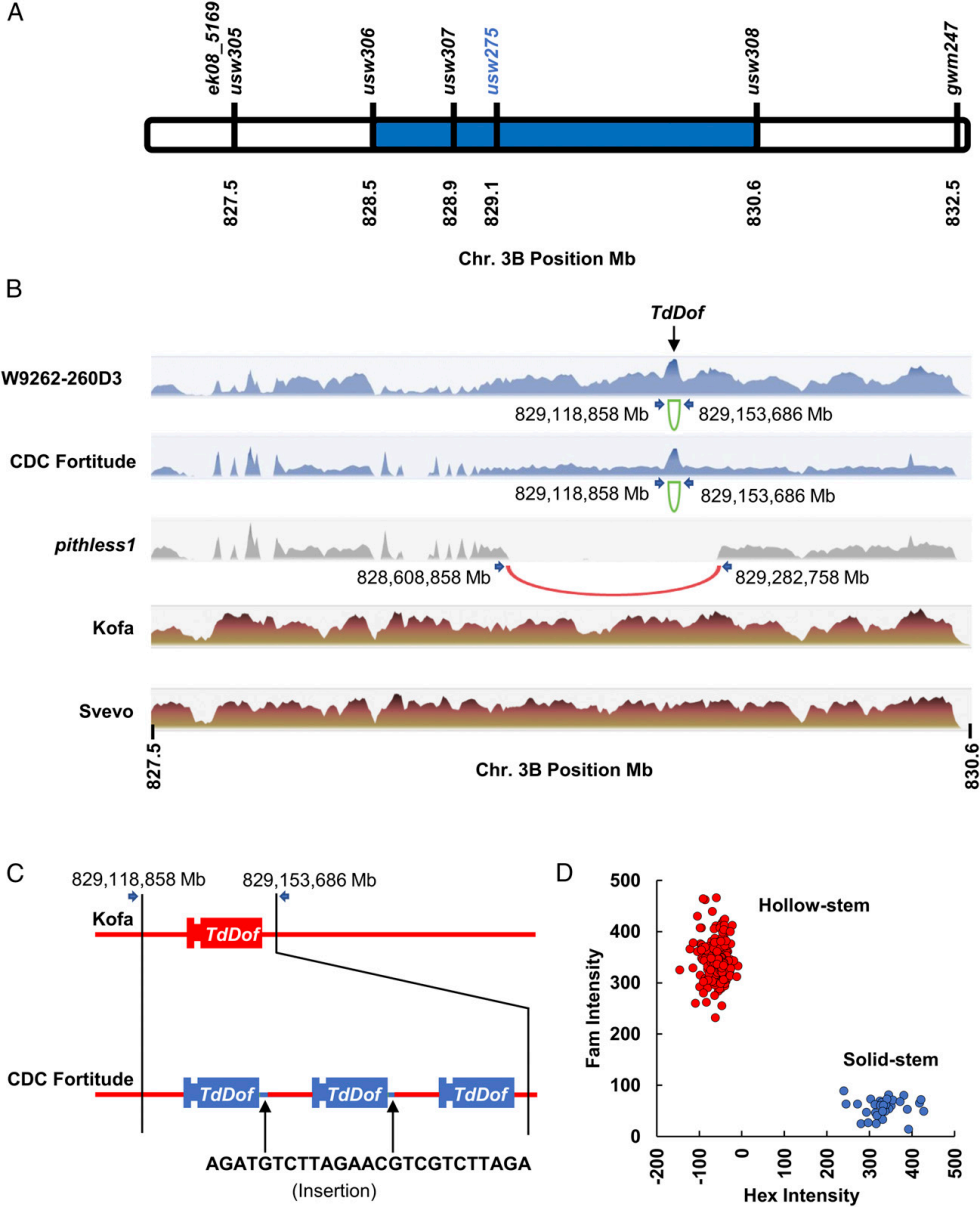
CNV of *TdDof* is associated with stem solidness in wheat. (*A*) Fine-map interval of *SSt1* in the Kofa/W9262-260D3 F_2_ population. The region between closest flanking markers *usw306* and *usw308* is shown in blue. (*B*) Chromium read coverage plots within the *SSt1* interval on chromosome 3B of five durum lines. The *y* axis of each coverage plot denotes the minimum and maximum read coverage for each line within the interval. GemCode molecule associations spanning structural variants are indicated by the green bars (CDC Fortitude, W9262-260D3) and red bar (*pithless1*). Positions are in megabases. (*C*) Diagram showing the organization of the *TdDof* CNV region in CDC Fortitude (solid-stemmed) and Kofa (hollow-stemmed) with CNV breakpoints indicated by the black bars. Three copies of *TdDof* (*TdDof1-3*) are present in CDC Fortitude vs. a single copy in Kofa. At the CNV breakpoint between *TdDof1-2* and *TdDof2-3*, a unique 25-bp insertion is present that is only found in solid-stemmed cultivars. (*D*) High-throughput KASP (kompetitive allele-specific PCR) marker *usw275* accurately distinguishes stems that are hollow (red) and solid (blue) in both bread and durum wheat.

To narrow the genomic and genetic interval of *SSt1*, we performed fine mapping using an F_2_ population derived from a cross between durum (tetraploid) lines Kofa (hollow-stemmed) and W9262-260D3 (solid-stemmed) with markers designed to target the *SSt1* region (*SI Appendix*, Table S1). Of 4,000 F_2_ lines examined, 24 critical recombinants were identified between flanking markers *ek08_5169* and *gwm247* (11). These lines were subsequently evaluated for stem solidness at maturity and used to position the *SSt1* gene on the physical map (Fig. 1*A*). The closest flanking markers to *SSt1* were *usw306* and *usw308*, which were anchored to physical positions 828.5 and 830.6 Mb, respectively, on chromosome arm 3BL in the durum wheat reference genome assembly of the cultivar Svevo (19). This allowed us to further narrow the previously identified interval from 13 Mb (11) to 2.1 Mb (Fig. 1*A*). Within this interval, 42 protein-coding genes are annotated in the Svevo genome (*SI Appendix*, Table S2).

### Copy Number Variation of a Putative Dof Transcription Factor (*TdDof*) Is Associated with Stem Solidness in Wheat.

To identify candidate genes within the *SSt1* fine-mapping interval, we generated an ethyl methanesulfonate mutant population derived from the solid-stemmed cultivar CDC Fortitude, which led to the selection of a hollow-stemmed mutant line “*pithless1*.” *pithless1 *was the only identified mutant exhibiting a complete loss of phenotypic expression of stem solidness. Within the *SSt1* region in *pithless1*, a large deletion spanning 673.9 kb was detected via coverage analysis of short-read Chromium whole genome sequencing (10× Genomics). This sequencing platform provides long-range information through short reads by incorporating barcode oligos (GemCode) from the originating DNA segment, thereby providing increased power to detect structural variants. The deletion in *pithless1 *was validated by the presence of GemCode molecule associations between both ends of the deletion breakpoints (Svevo 3B positions 828,608,858 and 829,282,758 Mb) (Fig. 1*B*). The deletion contains 11 protein-coding genes, which are annotated to encode the following proteins: a Werner syndrome-like exonuclease, disease resistance protein RPM1, vacuolar protein sorting protein 25, two metallothioneins, plant invertase/pectin methylesterase inhibitor superfamily protein G, 30S ribosomal protein S17, 30S ribosomal protein S19, SANT domain-containing protein 2 G, very-long-chain (3R)-3-hydroxyacyl-CoA dehydratase, and a Dof zinc finger protein (TdDof) (*SI Appendix*, Table S2). The hollow-stemmed phenotype of *pithless1 *and the deletion of these 11 genes suggest that the molecular determinant responsible for *SSt1* is located within this region.

In contrast to our observations in *pithless1*, we identified an increase in read coverage within the *SSt1* interval spanning a 34.8-kb interval (Svevo 3B positions: 829,118,858 and 829,153,686 Mb) in the solid-stemmed lines CDC Fortitude and W9262-260D3, indicating that additional copies of this segment are present in each of these lines (Fig. 1*B*). Importantly, this region of copy number variation (CNV) contains only one gene (*TRITD3Bv1G280530*) in Svevo that encodes a putative Dof zinc finger protein (*TdDof*) and is one of the 11 deleted genes in the *pithless1 *mutant (*SI Appendix*, Table S2). Analysis of the GemCode molecule associations spanning this region in CDC Fortitude and W9262-260D3 indicated that all copies of *TdDof* are arranged in tandem (Fig. 1 *B* and *C*). In contrast, visualization of the read data for the hollow-stemmed lines Svevo and Kofa indicated that a single copy of the region containing *TdDof* is present in these lines (Fig. 1*B*). The overlap between the deleted region in *pithless1 *and the CNV in solid-stemmed lines supports *TdDof* as a strong candidate for *SSt1*.

### Solid-Stemmed Cultivars Carry Three Identical Copies of *TdDof*.

To resolve the *TdDof* CNV region in solid-stemmed lines, we performed targeted sequencing and assembly of the region in CDC Fortitude following a modified CRISPR-Cas9 protocol from Oxford Nanopore Technologies. The reason we chose this approach was twofold; first, to fully resolve the tandem copies of *TdDof*, ultralong reads that span the CNV breakpoints are required, and, second, because of the size of the wheat genome, targeted sequencing of the *TdDof* region was a more efficient strategy. We generated an assembly of 230,823 base pairs (bp) that contained three copies of *TdDof* (*TdDof*_*1–3*_; *SI Appendix*, Fig. S1*A*), confirming the presence of two additional copies of *TdDof* in CDC Fortitude arranged in tandem orientation, each with an identical upstream putative regulatory region (Fig. 1*C* and *SI Appendix*, Fig. S1 *A* and *B*). A unique 25-bp insertion sequence (AGA​TGT​CTT​AGA​ACG​TCG​TCT​TAG​A) was identified at the insertion breakpoint between *TdDof*_*1*_ and *TdDof*_*2*_ and between *TdDof*_*2*_ and *TdDof*_*3*_ that was not present in any of the hollow-stemmed lines examined (Fig. 1*C* and *SI Appendix*, Fig. S1*B*). We used Droplet Digital PCR (ddPCR), which provides absolute quantification of target DNA or RNA molecules, to confirm the presence of three copies of *TdDof* and two copies of the unique 25-bp insertion in CDC Fortitude compared to the single copy of *TdDof* in Kofa and no copies of the insertion sequence (*SI Appendix*, Fig. S1*C*). We also developed a dominant PCR marker to detect the CNV region and validated it in three lines derived from common representative sources of stem solidness: Golden Ball, Biodur, and S-615 (*SI Appendix*, Fig. S1*D*). These results suggest that increased copy number of *TdDof* is associated with the *SSt1* solid-stem phenotype.

*TdDof* is located between positions 829,151,607 and 829,152,953 on chromosome 3B in the Svevo reference genome (*SI Appendix*, Table S2). The gene contains two exons and one intron (*SI Appendix*, Fig. S2), and the coding sequence (CDS) of 1,134 nucleotides encodes a predicted protein of 377 amino acids (*SI Appendix*, Fig. S2). The predicted protein sequence encoded by *TdDof* is identical between hollow- and solid-stemmed lines. In solid-stemmed lines, each *TdDof* copy (*TdDof*_*1–3*_) encodes an identical protein. The predicted protein contains a zinc finger Dof domain (IPR003851) between amino acid residues 110 and 164 (*SI Appendix*, Fig. S2). The Dof domain is a highly conserved DNA-binding domain that is specific to a family of transcription factors observed in vascular plant species.

### Overexpression of *TdDof* Induces Stem Solidness in Kronos and *pithless1*.

**Fig. 2.**
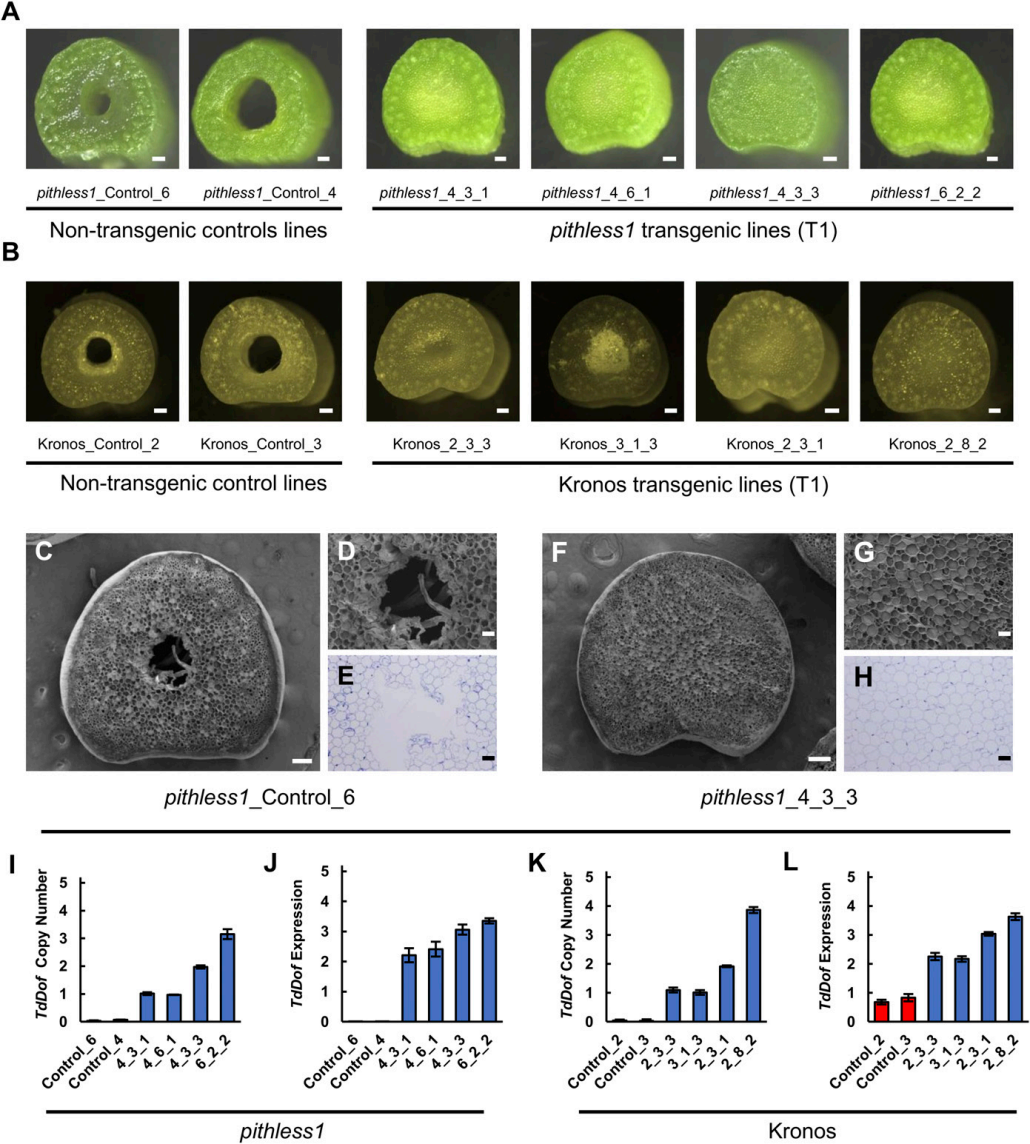
Transgenic expression of *TdDof* induces stem solidness in hollow-stemmed lines *pithless1 *and Kronos. Cross-sections of the second internodes of nontransgenic (*Left*, hollow stem) and *TdDof* overexpression T1 transgenic lines (*Right*, solid stem) derived from *pithless1 *(*A*) and Kronos (*B*) at Zadoks stage 34. Scanning electron microscopy images of stem cross-sections showing the detailed stem anatomy in *pithless1_*Control_6 (nontransgenic line) (*C* and *D*) compared *TdDof* overexpression T1 transgenic line *pithless1*_4_3_3 (*F* and *G*). Light microscopy images of stem cross-sections (*E* and *H*). In the high-magnification view of the central regions of these sections (*D*, *E*, *G*, and *H*), parenchyma cells in and surrounding the central pith region are intact and more uniform in size in the *TdDof* overexpression T1 transgenic lines (*G* and *H*), whereas, in nontransgenic control lines in the comparable pith region, the cells are irregular or broken and collapsed (*D* and *E*). (Scale bars: *A*–*C* and *F* = 2 mm; *D*, *E*, *G*, and *H* = 500 µm.) Molecular analysis via ddPCR of *TdDof* transgene copy number and gene expression in independent T1 transgenic lines of *pithless1 *(*I* and *J*) and Kronos (*K* and *L*). Error bars denote SD across replicates (*I*–*L*). The same lines imaged in *A* and *B* were selected for molecular studies.

To investigate the phenotypic effects of increased *TdDof* gene expression, we generated a series of independent transgenic lines in the hollow-stemmed wheat lines *pithless1 *and Kronos that overexpress the *TdDof* CDS under control of the rice *ACTIN* promoter. All T_0_ lines were analyzed for *nptII* copy number to confirm the presence of the *TdDof* transgenic construct. Both the *pithless1 *and Kronos controls regenerated from calli carried 0 transgene copies, whereas the transgenic lines carried between 1 and 4+ copies of the *TdDof* construct (*SI Appendix*, Table S3). For the *pithless1*-derived transgenic plants, 9 out of 10 T_0_ lines had the maximum level of stem solidness, in contrast to the *pithless1 *control which had hollow stems (*SI Appendix*, Table S3). For the Kronos-derived transgenic plants, 27 out of 30 T_0_ lines had stems that were solid compared to the Kronos control, which had hollow stems (*SI Appendix*, Table S3). To further validate these observations, we advanced the T_0_ plants in both backgrounds to the T_1_ generation. Homozygous T_1_ plants were phenotypically similar to the controls, except for the accumulation of pith. Some variation in the color of pith was observed between transgenic lines, which ranged between green and white. Importantly, the T_1_ progeny maintained the stem solidness phenotype that was observed in the T_0_ generation (Fig. 2 *A*–*H*). The increased copy number of the *TdDof* constructs in the transgenic lines was associated with increased *TdDof* transcript levels (Fig. 2 *I*–*L*). Therefore, overexpressing *TdDof* in two independent genetic backgrounds (Kronos and the *pithless1 *mutant) resulted in stem solidness, providing strong evidence that *TdDof* is the *SSt1* locus is a causal genetic factor of stem solidness.

### Gene Expression Signatures Identify Candidate Pathways Involved in *TdDof*-Mediated Stem Solidness.

**Fig. 3.**
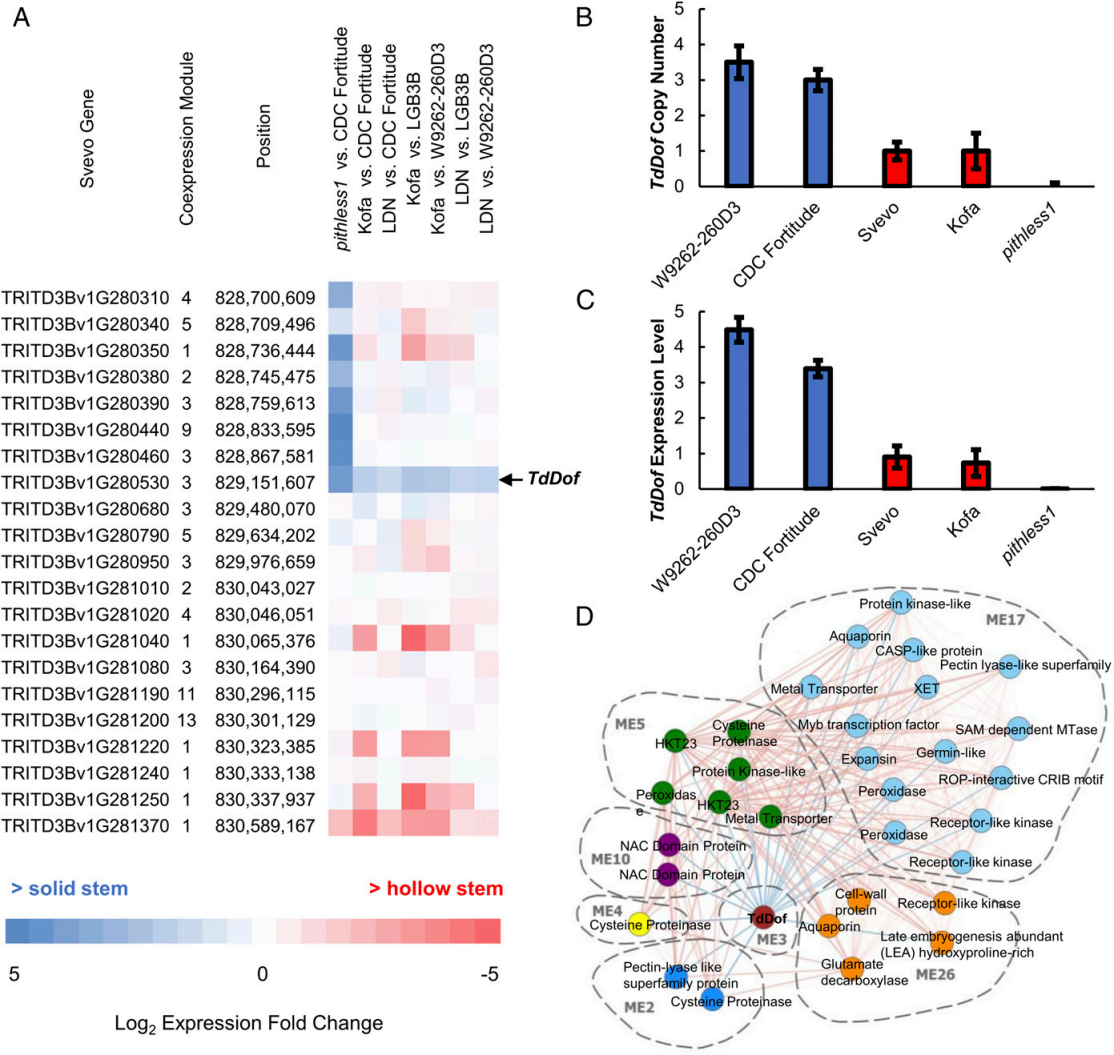
*TdDof* expression and mediated stem solidness pathways are associated with CNV. (*A*) Heat map of high-confidence genes that were expressed inside the *SSt1* fine-mapping interval. Pairwise comparisons (hollow vs. solid) are presented as a heat map of values as Log_2_ fold-change (Log_2_ FC), where blue indicates higher expression was detected in the solid-stemmed line, and red indicates higher expression was detected in the hollow-stemmed line. For each gene, its corresponding coexpression module is indicated. (*B*) The ddPCR validation of *TdDof* gene copy number (calculated as the ratio of absolute concentration [copies per microliter] between each sample and the single-copy reference control gene *TaActin*), with multiple copies in solid-stemmed W9262-260D3 and CDC Fortitude (blue bars), a single copy in hollow-stemmed Svevo and Kofa, and no copies in *pithless1 *(red bars). (*C*) *TdDof* gene expression levels, with greater expression in solid-stemmed lines W9262-260D3 and CDC Fortitude (blue bars), lower expression in hollow-stemmed lines Svevo and Kofa, and no expression in *pithless1 *(red bars). (*D*) Gene expression network plot. Modules (MEs) were defined as clusters of interconnected genes, and each module was assigned a unique color. Lines connecting genes in the network plot indicate correlation in expression patterns across samples. A positive correlation is denoted by red line, whereas a negative correlation is denoted by a blue line.

We performed gene expression profiling of six durum wheat lines with hollow- and solid-stemmed types to identify differentially expressed genes (DEGs) during early internode elongation (Zadoks stage 32). This is typically when the first sign of pith breakdown begins to appear in hollow-stemmed cultivars. The experiment was performed in three replications for each line. Within the *SSt1* interval, 21 of the 42 genes were expressed (raw mean read count >15), including a cluster of eight genes (including *TdDof*) that were differentially expressed between *pithless1 *and CDC Fortitude (Fig. 3*A*). All eight genes are contained within the *pithless1 *deletion, and thus were not expressed in the mutant. Of the eight genes, only *TdDof* was consistently differentially expressed (up-regulated in solid-stemmed lines) across all pairwise comparisons (Fig. 3*A*). These results indicate that *TdDof* expression is associated with stem solidness, and its gene expression pattern is positively associated with the genomic copy number of *TdDof* (Fig. 3 *B* and *C*).

To investigate genome-wide regulatory pathways involved in stem solidness, we performed weighted gene coexpression network analysis (Fig. 3*D* and Table 1). In a pairwise comparison between *pithless1 *and CDC Fortitude, only 195 DEGs were identified, which were distributed across all chromosomes (adjusted *P* < 0.01) (Dataset S1). Of these, we identified 12 “primary” genes that were codifferentially expressed in all hollow- by solid-stemmed pairwise comparisons (Table 1). These genes have been annotated to encode the following proteins: peroxidase (*TRITD1Bv1G046830*), two NAM/ATAF1/2/CUC2 (NAC) domain proteins (*TRITD2Av1G226090* and *TRITD2Bv1G188540*), three cysteine proteinases (CEP, *TRITD3Av1G213290*, *TRITD3Bv1G195680* and *TRITD3Bv1G196170*), a Dof zinc finger protein, *TdDof* (*TRITD3Bv1G280530*), two metal transporters (*TRITD4Av1G000570* and *TRITD4Bv1G174140*), a pectin lyase-like superfamily protein (*TRITD6Av1G021640*), and two HKT23 transporters (*TRITD7Av1G226240* and *TRITD7Bv1G172380*). The expression of *TdDof* in the solid-stemmed lines was negatively correlated with the expression of all 11 other primary genes (Table 1). Notably, this list includes three *CEP* genes, and two *NAC* transcription factor genes whose orthologs have been implicated in PCD in other species (Table 1), and a pectin lyase-like superfamily protein putatively involved in cell wall degradation.

**Table 1.** List of genes identified in coexpression network analysis

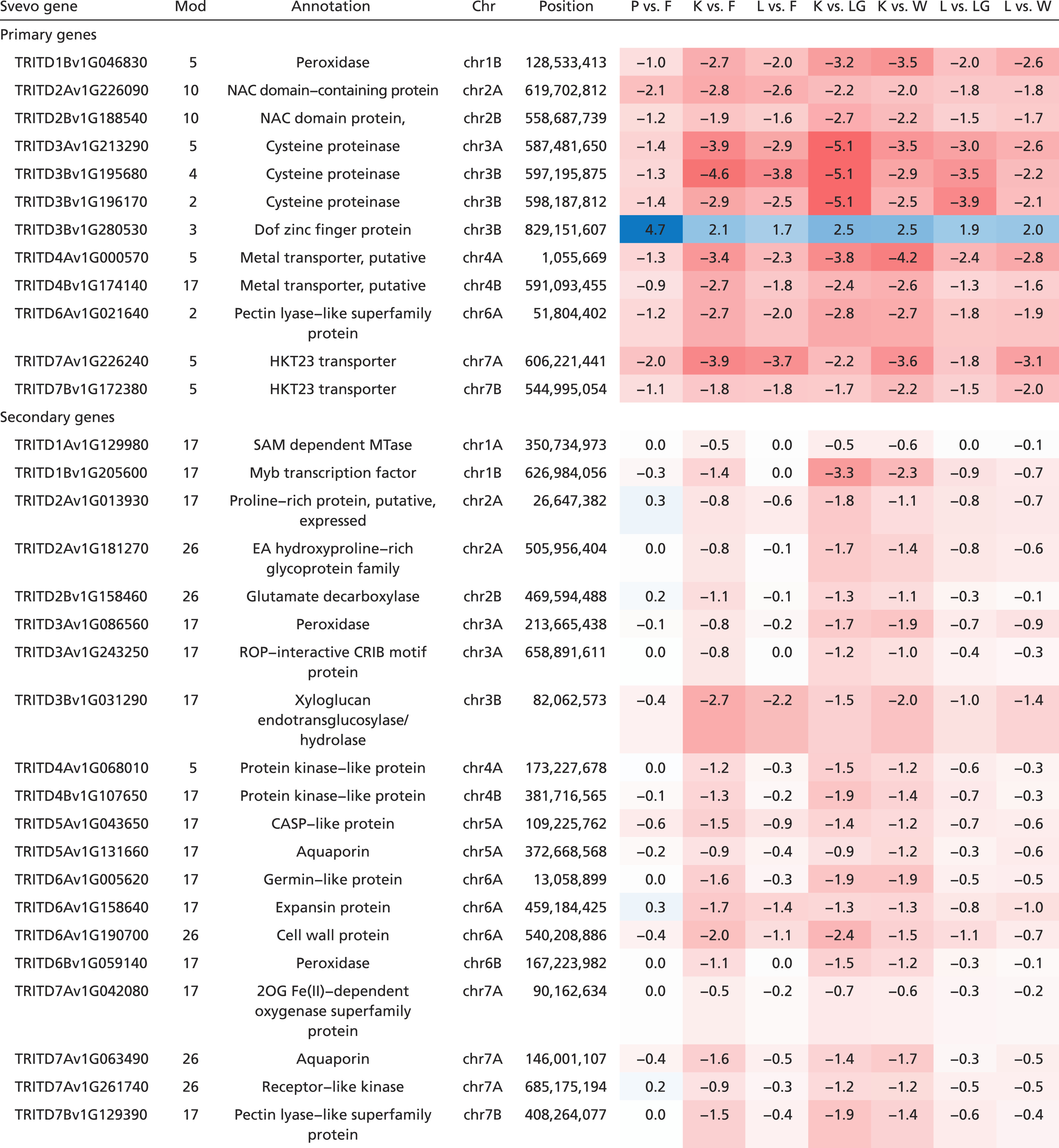

The table presents 12 primary genes that met the criteria of differential expression across all hollow- vs. solid-stemmed comparisons. This list was used to identify secondary genes that were correlated in their expression across samples with at least one of the primary DEGs. The table lists the gene accession from the Svevo annotation, coexpression module (Mod), Svevo gene description, chromosome (Chr), and position. Values on the right of the table are log_2_ expression fold-change values for each hollow by solid comparison. Positive values indicate expression was greater in the solid-stemmed line denoted by blue shading, and negative values indicate expression was greater in the hollow-stemmed line denoted by red shading. Genotypes used in pairwise comparisons are labeled as follows: P, *pithless1*; F, CDC Fortitude; L, Langdon; LG, Langdon-GB-3B; K, Kofa; W, W9262-2603.

Next, we used the list of “primary” genes to extract “secondary” genes from the weighted gene coexpression network based on their correlated expression patterns (Table 1 and Dataset S1). Using this approach, we constructed a subnetwork consisting of 32 genes from seven different coexpression modules (Fig. 3*D*). Secondary genes were annotated as being putatively involved in cell wall modification/degradation, transcriptional regulation, central metabolism, and signal transduction (Table 1). Taken together, our studies indicate that higher levels of *TdDof* expression in solid-stemmed cultivars is correlated with the suppression of genes involved in several processes including cell wall modification/degradation, and the regulation and execution of PCD.

### Developmental and Metabolite Analyses Identify Structural and Metabolomic Consequences of *TdDof-*Mediated Stem Solidness.

**Fig. 4.**
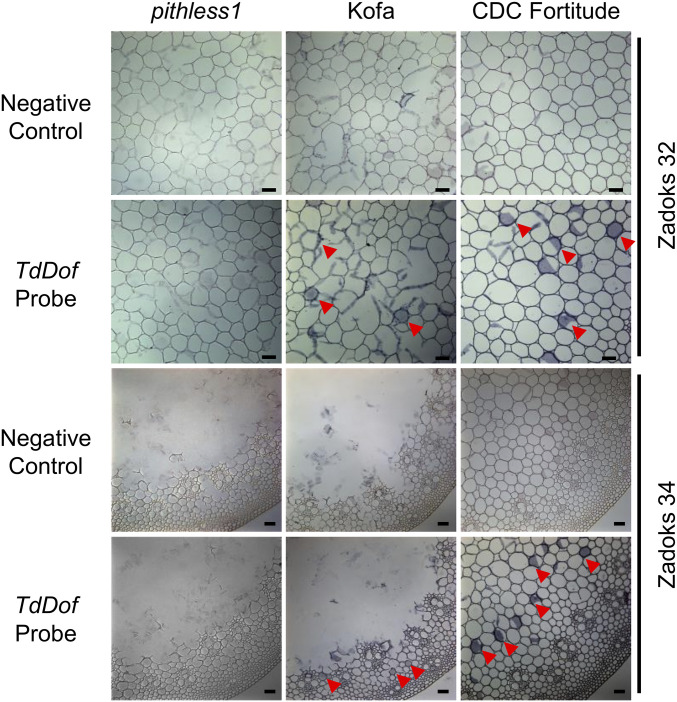
In situ PCR demonstrating enrichment of *TdDof* messenger RNA in internode parenchyma cells in *pithless1*, Kofa, and CDC Fortitude. Purple staining (labeled by red arrows) indicates the presence of in situ PCR amplified *TdDof* transcripts. Representative micrographs of internode cross-sections from Zadoks stages 32 and 34 show similar high expression levels of *TdDof* in parenchyma cells in which the negative controls were generated under the same conditions but with the reverse transcription steps omitted. Three panels from *Left* to *Right* represent internode tissues from *pithless1*, Kofa, and CDC Fortitude. (Scale bars = 200 μm.)

To address the developmental pathways involved in the distinct stem solidness phenotypes in wheat, we performed a detailed comparison of stem pith anatomy between CDC Fortitude and *pithless1 *using stem cross-sections sampled at Zadoks stage 32 (*SI Appendix*, Fig. S3). Light and electron microscopy revealed clear differences in the central pith parenchyma cells of *pithless1 *compared to CDC Fortitude, including irregular, broken, and collapsed parenchyma cells and cell wall buckling. These anomalies in pith parenchyma cell size and shape prompted us to analyze the spatial expression pattern of *TdDof* in the stem for any potential associations with these morphological changes. We performed in situ PCR using stem cross-sections collected at Zadoks stages 32 and 34, from *pithless1*, Kofa, and CDC Fortitude (Fig. 4). *TdDof* transcripts were predominantly expressed in pith parenchyma cells of Kofa and CDC Fortitude during second internode formation (Fig. 4). During stem elongation, hollow pith gradually formed in Kofa (Zadoks stage 34), while *TdDof* was only expressed in intact parenchyma cells (Fig. 4). By contrast, the expression pattern of *TdDof* in CDC Fortitude at Zadoks stage 34 remained constant and was similar to Zadoks stage 32, which was associated with reduced cell death and pith degradation (Fig. 4). As expected, no signals above the background level were detected in *pithless1*, which is consistent with the deletion of *TdDof* (Fig. 4).

**Fig. 5.**
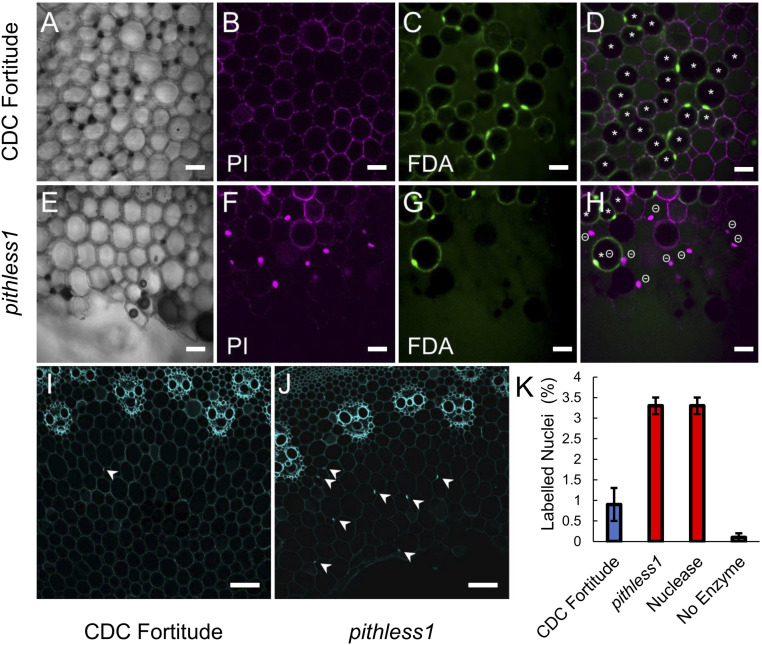
Cell viability in the pith parenchyma cells of CDC Fortitude and *pithless1 *stems. Stem cross-sections from the second internodes of CDC Fortitude (*A*–*D* and *I*) and mutant *pithless1 *(*E*–*H* and *J*) plants viewed as single frame images. Viability staining of pith cells viewed by confocal laser-scanning microscopy phase contrast image (*A* and *E*), PI signal (*B* and *F*), FDA signal (*C* and *G*), and PI and FDA merged view (*D* and *H*). Parenchyma cell walls stained with PI in CDC Fortitude (*B*) and *pithless1 *(*F*), with fluorescent nuclei indicating penetration of the dye into cells surrounding the hollow pith of *pithless1 *stem sections. Penetration and conversion of nonfluorescent FDA stain into green fluorescent fluorescein by CDC Fortitude (*C*) and *pithless1 *(*G*) pith parenchyma cells. The * symbol indicates a putatively viable cell, with bright FDA signal around a dark vacuole, and the Θ symbol indicates a cell considered to be dead, with PI-positive nucleus. The viability status of cells without labels is undetermined. TUNEL assay for DNA fragmentation in CDC Fortitude (*I*) and *pithless1 *(*J*) stems, as viewed by confocal laser scanning microscopy. Arrowheads indicate positive nuclear signals in pith cells. (*K*) Quantification of TUNEL assay results for three biological replicates of CDC Fortitude (Fort), *pithless1 *(P1), positive control using TACS nuclease treatment (+), and unlabeled experimental control (−), with the labeled nuclei per the total number of pith parenchyma cells plotted as the mean percentage ±SD. (Scale bars: *A*−*H* = 20 µm; *I* and *J* = 100 µm.)

To investigate the potential cause of cell collapse in the hollow stems of *pithless1*, we compared cell viability in the pith parenchyma cells at Zadoks stage 32 in the stem internodes of CDC Fortitude and *pithless1 *using two complementary cell viability stains, fluorescein diacetate (FDA) and propidium iodide (PI), followed by confocal laser scanning microscopy (Fig. 5 *A*–*H*). PI stains only nuclei from dead cells with ruptured cell membranes, making them appear fluorescent, whereas nuclei from living cells are not stained by this dye. FDA stains viable, living cells producing nuclear and nucleocytoplasmic signals around a dark vacuole where stain is quenched. In our analysis, the absence of nuclear staining by PI alone was not used to determine the viability status of a cell, as select nuclei may be out of the optical plane or excluded by specimen preparation. Thus, putatively viable cells were defined as FDA stain positive, while dead or dying cells were defined by a PI-stained nucleus. The occasional observation of a cell with both FDA-positive staining and a PI-stained nucleus was defined as dead. *pithless1 *stem sections stained with PI had abundant fluorescent nuclei in parenchyma cells compared to CDC Fortitude, when the same Z-stack thickness and step size were used in image collection, pointing to increased PI uptake and cell death in *pithless1 *(Fig. 5 *A*, *B*, *E*, and *F*). In support of the PI staining patterns, a lower proportion of pith parenchyma cells was stained by FDA, indicating the presence of fewer living, viable cells among the cells surrounding the hollow pith of *pithless1 *stems relative to CDC Fortitude (Fig. 5 *C*, *D*, *G*, and *H*). In addition to showing that cell viability was reduced in the pith parenchyma cells of *pithless1 *stems, these data indicate that the cells in *pithless1 *were undergoing cell death prior to their collapse (Fig. 5 *D* and *H*). To assess the potential mechanism underlying cell death in *pithless1 *pith parenchyma cells, we performed a terminal deoxynucleotidyl transferase (TUNEL) assay to quantify in situ DNA fragmentation in CDC Fortitude and *pithless1*, as examined by confocal laser scanning microscopy (Fig. 5 *I* and *J* and *SI Appendix*, Figs. S4 and S5). When the same Z-stack thickness and step size were used to ensure nuclei at multiple depths were captured, there was an approximately threefold increase in the number of positive nuclear signals in the pith cells of *pithless1 *compared to CDC Fortitude, supporting the notion that PCD occurred in parenchyma cells prior to cell collapse and cavity formation in the pith (Fig. 5*K*). Together, these results strongly support the notion that the reduction or loss of *TdDof* expression is associated with the activation of PCD of pith parenchyma cells, thereby inducing the formation of a hollow pith within the stem.

To explore the differences in the biochemical compositions between hollow- and solid-stemmed lines, we performed metabolite profiling during three stages (Zadoks stages 32, 33, and 34) of stem development. Statistical analyses for pairwise sample comparisons are presented (Dataset S2). CDC Fortitude showed strong differences in glutathione metabolism relative to *pithless1 *and Kofa, both of which accumulated high amounts of glutamate and gamma-glutamyl acids; these compounds function as strong osmolytes and accumulate in tissues undergoing controlled desiccation (*SI Appendix*, Fig. S6 and Dataset S2). By Zadoks stage 34, the levels of 60 metabolites differed in *pithless1 *and Kofa compared to CDC Fortitude, pointing to differences in biochemical activity between hollow- and solid-stemmed types. The levels of most amino acids and their metabolites were higher in *pithless1 *than CDC Fortitude, whereas the levels of almost all lipids were lower in these lines. The levels of nitrogen-rich compounds asparagine, glutamine, spermidine, and allantoin were higher in *pithless1 *than in CDC Fortitude, with levels up to >100-fold higher in the mutant. The levels of several compounds in the glutathione cycle were much higher in *pithless1 *than in CDC Fortitude (5-oxoproline, the *gamma*-glutamyl amino acids), as were the levels of amino acids that function as strong osmolytes (proline, trigonelline). WSC (1-kestose, glucose, fructose, sucrose) content in the stem varied among lines and developmental stages. Notably, CDC Fortitude accumulated high levels of 1-kestose by Zadoks stage 34. At this stage, the sucrose content was similar between CDC Fortitude and Kofa but significantly lower in *pithless1 *(*SI Appendix*, Fig. S6 and Dataset S2). Taken together, these results indicate that the hollow-stemmed lines Kofa and *pithless1 *have increased levels of osmolytes (indicators of controlled desiccation and PCD), as well as differences in WSC content compared to CDC Fortitude.

### Developing a DNA Marker for Breeding Solid-Stemmed Cultivars.

A high-throughput universal marker for the solid-stemmed trait that can be used in both durum and bread wheat germplasm has remained elusive. We previously described the marker *usw204*, which is currently used in some breeding programs (20); however, this marker scores hexaploid and tetraploid cultivars inversely (*SI Appendix*, Fig. S7*A*). To overcome this limitation, we mined the Chromium sequence datasets to call single-nucleotide polymorphisms and designed additional markers. We identified a region spanning position 828.9 Mb to 829.6 Mb on chromosome 3B of the Svevo assembly in which the allelic states of solid-stemmed hexaploid and tetraploid cultivars were identical (*SI Appendix*, Fig. S7*B*). This interval is consistent with the fine-mapped interval in durum wheat that contains the *SSt1* gene *TdDof.* We successfully developed a marker (*usw275*) tightly linked (<4.3 kb) to *TdDof* that produces accurate results in both bread and durum wheat and, most importantly, is amenable to high-throughput genotyping and the tracking of stem solidness in breeding programs (Fig. 1*D* and *SI Appendix*, Table S4).

## Discussion

Here, we identified *TdDof* (*TRITD3Bv1G280530*), which encodes a putative Dof (DNA binding with one finger) protein, as the causal gene conferring stem solidness corresponding to *SSt1* on chromosome arm 3BL. We confirmed this via transgenic overexpression of *TdDof* in two independent hollow-stemmed durum wheat genotypes which yielded transformants with solid stems. Although all cultivars we examined carry the genetic sequence to encode identical functional TdDof proteins irrespective of stem type, our findings indicate the solid-stemmed phenotype is caused by increased expression of the *TdDof* gene. Three identical copies of *TdDof* in solid-stemmed durum wheat are tandemly arranged within a 34.8-kb triplicated segment within the *SSt1* locus. The expression of *TdDof* during early stem elongation is positively correlated with its genomic copy number, and higher expression likely induces the solid-stem phenotype. Given that the upstream putative regulatory region is present for each of the three *TdDof* copies, coupled with an approximate threefold increase in gene expression, it is likely that all three copies are expressed in solid stemmed cultivars. However, we cannot rule out the possibility that one or more copies of *TdDof* contribute more to the observed expression levels.

Stem solidness is a trait that has been deployed in wheat to control devastating outbreaks of the WSS in North America. The conventional sources used to introgress stem solidness into wheat were the Portuguese landrace S-615 (hexaploid bread wheat), the South African landrace Golden Ball (tetraploid durum wheat), and the German cultivar Biodur (tetraploid durum wheat) (12–14). Despite their diverse global origins and differing ploidy levels, the unique *TdDof* CNV signature is found in all three of these sources of stem solidness. This finding, combined with an accurate DNA marker that predicts stem solidness within both wheat species, not only supports that *TdDof* is responsible for stem solidness in both durum and bread wheat but also indicates that stem solidness originated from a common genetic progenitor. However, the original source has not been identified, and it remains to be determined whether stem solidness in wheat originated pre- or postdomestication.

*TdDof* is part of a large family of plant-specific transcription factors that were first identified in maize (21). Since their discovery, Dof transcription factors have been shown to regulate a wide range of developmental and physiological processes, including carbon and nitrogen assimilation (22), hormone signaling (23), light responses (24), seed germination and development (25), flowering time (26–28), pollen development (29), shoot branching (30), interfascicular cambium/vascular tissue development (31), biotic stress tolerance (32), and drought and salt tolerance (28). There are 62 Dof genes in durum wheat and 116 in bread wheat. The recent release of the first genome sequences for durum and bread wheat (19, 20), along with high-quality gene models, has set the stage for unraveling the functions of this diverse gene family and their associated regulatory functions. Here, we demonstrated that *TdDof* controls stem architecture, and leads to reduced expression of genes putatively involved in PCD such as *NAC* (*TRITD2Av1G226090* and *TRITD2Bv1G188540*) and *CEP (TRITD3Av1G213290*, *TRITD3Bv1G195680*, and *TRITD3Bv1G196170*). Furthermore, *TdDof* may also impact response to oxidative stress (peroxidase: *TRITD1Bv1G046830*), metal ion transport (*TRITD4Av1G000570 and TRITD4Bv1G174140*), cell wall modification (pectin lyase-like superfamily: *TRITD6Av1G021640*) and cation transport (*HKT2*: *TRITD7Av1G226240* and *TRITD7Bv1G172380*).

Our findings suggest that *TdDof* may impact PCD pathways through direct or indirect regulation of NAC and CEP genes, as supported by histochemical evidence of PCD of the pith parenchyma in hollow-stemmed cultivars during the early stages of stem elongation. PCD is an important regulatory mechanism used by plants to eliminate unwanted cells, a process required for normal plant growth and development (33, 34). One example of PCD is the hypoxia-driven occurrence of pith autolysis, a widespread phenomenon in many plant species that is necessary for eliminating pith parenchyma cells and forming aerenchyma to facilitate gas exchange within the stem (35). In the current study, we provided several key lines of evidence suggesting PCD is associated with stem hollowness in wheat. Gene coexpression analysis identified two NAC genes on chromosomes 2A and 2B and three CEP genes on chromosomes 3A and 3B that were strongly up-regulated in hollow stems where *TdDof* expression was low. In *Arabidopsis*, the close ortholog to *TRITD2Av1G226090*, *TRITD2Bv1G188540* is the gene *KIRA1*, whose expression was implicated as a key regulator of PCD-associated genes in the stigma (36). Recent studies identified the *D* gene (*Sobic.006G147400*) in sorghum (*Sorghum bicolor* L.), encoding an NAC transcription factor that functions as a transcriptional switch to regulate PCD in stems (37, 38). The *D* gene activates various cell death-related enzymes, including the direct activation of the cysteine protease *CEP1* (37, 38). The most closely related genes to the sorghum *D* gene in the durum wheat annotations share 86% protein sequence similarity with NAC protein TRITD2Bv1G188540 encoded on chromosome 2B and 66% sequence similarity with NAC protein TRITD2Av1G226090 encoded on chromosome 2A, indicating that these proteins are orthologous between sorghum and wheat. Cysteine protease genes, including *CEP1* that is regulated by the sorghum *D* gene, belong to a large family of enzymes that are ubiquitous across plants, animals, and microbes. These enzymes function downstream of transcription factors and are involved in protein degradation and PCD (39, 40). The *Arabidopsis* ortholog, *CEP1,* has been implicated in PCD during xylem development (41). Along with *CEP1*, several other known protease genes are often coexpressed in cells that are actively undergoing PCD (42–44). Given the strong negative correlation we observed between the expression of *TdDof* and PCD-related genes, this observation suggests that NAC and CEP genes could be target genes that are negatively regulated by *TdDof*. Nevertheless, the direct link between *TdDof* expression and the occurrence of PCD remains unclear and warrants further investigation.

We also obtained histochemical evidence suggesting the cells in *pithless1 *undergo PCD prior to collapsing into the culm lumen. However, unlike stems in sweet and juicy sorghum cultivars, whose pith remains largely intact, the complete collapse of the pith in most hollow-stemmed wheat occurs during the early stages of stem elongation. Despite this difference, our findings suggest that the physiological processes identified in sorghum (37, 38) are likely similar to the processes that occur in hollow-stemmed wheat cultivars. Targeting genes involved in pith PCD, including those identified in this study such as the NAC and CEP genes, might represent a new avenue for enhancing the expression of stem solidness in crop species. For example, because solid stems have higher mass per unit length than hollow stems, this could have positive implications for biomass partitioning and may be useful in the production of bioethanol. If PCD pathways are conserved within the stem between species, genetic engineering involving *TdDof* could be one strategy to increase accessible WSC content in other monocot crops such as switchgrass (*Panicum virgatum* L.), which is used in the biofuel industry for ethanol production (45). Increasing WSC content in straw could increase the extraction efficiency of ethanol while lowering the amount of enzymatic pretreatment required during processing (46).

Our findings lay the foundation for future research aimed at optimizing the expression of stem solidness, which could have additional downstream agricultural and industrial applications. An important breeding target should be to maximize pith production when female WSS are actively laying eggs, which would provide maximum levels of resistance to this insect. However, it may be beneficial to begin degrading the pith during grain filling to enhance water and WSC remobilization capacity to the developing grain, particularly during periods of abiotic stress. Drought poses the single largest constraint to crop production in many parts of the world and is expected to become more widespread due to the changing global climate. Increasing the capacity of the pith to store water and WSC for later remobilization under periods of abiotic stress has been suggested (8), but remains largely unexplored as a breeding target for enhancing drought stress tolerance. Indeed, in the current study, we observed the accumulation of higher levels of the fructan 1-kestose in the stems of CDC Fortitude relative to the hollow-stemmed controls. Consistent with this possibility, during periods of drought, hexose sugars from hydrolyzed fructans reduce leaf intracellular water potential to maintain turgor and to promote continued leaf expansion (4). Higher sugar concentrations also increase the chemical potential of water, which lowers the freezing point in nonfrozen tissues or promotes the melting of ice in frozen tissues (47), making it possible for plants with solid stems to have better cold stress tolerance than hollow-stemmed plants.

In summary, we determined that *TdDof* controls pith development in wheat. We propose that CNV of *TdDof* drives increased gene expression and the solid-stemmed phenotype. When we deleted all copies of *TdDof* in the solid-stemmed parent CDC Fortitude to generate the mutant *pithless1*, we observed a corresponding loss of the solid-stemmed phenotype associated with this deletion. Adding *TdDof* back into the *pithless1 *genetic background via exogenous overexpression restored the solid-stemmed dominant phenotype of this mutant. This gain of function was also observed in the unrelated genetic background of hollow-stemmed cultivar Kronos. Our newly developed molecular marker *usw275* will be useful for tracking *TdDof*-mediated stem solidness to facilitate research and breeding efforts. Our results also provide insight into the downstream pathways that may be regulated by *TdDof*, particularly the repression of PCD as a plausible mechanism for stem solidness.

## Materials and Methods

Details regarding sample preparation, experimental procedures, and data analysis, along with the associated references, are presented in *SI Appendix*, *SI Materials and Methods*. The 10× Genomics Chromium whole-genome sequencing data and Oxford Nanopore and RNA-seq (Illumina) data have been submitted to Sequence Read Archives at National Center for Biotechnology Information (NCBI) under BioProject accession number PRJNA630287 (48).

## Supplementary Material

Supplementary File

Supplementary File

Supplementary File

## Data Availability

Sequencing data have been deposited in NCBI (PRJNA630287) (48). All study data are included in the article and *SI Appendix*.
