# Supplementary material for: Copy number variation of TdDof controls solid-stemmed architecture in wheat
Source: Proc Natl Acad Sci U S A. 2020 Oct 30;117(46):28708–18. doi: 10.1073/pnas.2009418117 (PMC7682410; doi:10.1073/pnas.2009418117)
Supplement: Supplementary File [file pnas.2009418117.sapp.pdf]

PNAS
www.pnas.org

Supplementary Information for

Copy number variation of *TdDof* controls solid-stemmed architecture in wheat

Kirby T. Nilsen[1,2†], Sean Walkowiak[1,3†], Daoquan Xiang[4], Peng Gao[4], Teagen D. Quilichini[4], Ian R. Willick[1], Brook Byrns[1], Amidou N'Diaye[1], Jennifer Ens[1], Krystalee Wiebe[1], Yuefeng Ruan[5], Richard D. Cuthbert[5], Melanie Craze[6], Emma J. Wallington[6], James Simmonds[7], Cristobal Uauy[7], Raju Datla[4], and Curtis J. Pozniak[1]

Corresponding Author: To whom correspondence should be addressed: Curtis J. Pozniak, curtis.pozniak@usask.ca

This PDF file includes:
Supplementary information text
Figures S1 to S7
Tables S1 to S4
Supplementary Dataset 1 to 2
References for SI

www.pnas.org/cgi/doi/10.1073/pnas.2009418117

Materials and Methods

Exome capture and bulked segregant analysis. Bulked segregant analysis (BSA) was performed on doubled haploid (DH) lines from a mapping population derived from the cross Kofa/W9262-260D3 (1). Stem solidness in this cross is derived from W9262-260D3. DNA was extracted and pooled from 20 solid-stemmed lines (*SSt1*+ bulk) and 20 hollow-stemmed lines (*SSt1*- bulk). The DNA was enriched to obtain gene-coding regions using the wheat exome capture array according to the procedures outlined in (2). High-throughput sequencing was performed on the Illumina HiSeq2500 platform with 2 x 100 bp paired-end chemistry. Raw sequence reads were processed with Trimmomatic v0.32 (3), and processed reads were aligned to the genome assembly of the durum wheat cv. Svevo using Novoalign v3.02.05 (Novocraft Technologies Sdn Bhd). Duplicate read mappings and improper read pairs were removed using Picard-Tools. SNP variants were called using the SAMtools v1.2.1 (4) mpileup command. Filters were applied requiring each bulk to be homozygous and to carry a different allele from the other. SNPs spanning the *SSt1* interval were converted to KASP-based markers for the fine mapping experiments using Primer3 (5) software.

Fine mapping. We developed an F2 fine-mapping population segregating for stem solidness from the same parents used in the DH population (Kofa/W9262-260D3). Approximately 4,000 individual F2 plants were screened with flanking markers *ek08_5169* and *gwm247,* as defined in a previous study (1). Lines showing recombination events between these markers were selected and screened with a series of KASP markers developed from exome capture BSA using the approach described above. Plants were grown to maturity and rated for stem solidness using a 1 to 5 visual scale (1). Stem solidness is a dominant trait, making it impossible to phenotypically distinguish between a heterozygous and homozygous carrier. Therefore, in some cases, it was necessary to screen F2-derived F3 lines to confirm the allelic state at *SSt1* in the F2. The genetic map was visualized using MapChart software (6). The map was compared to the anchored marker positions in the Svevo (7) and Chinese Spring (8) genome assemblies.

Generating the CDC Fortitude mutant population. An ethyl methanesulfonate (EMS) mutant population was created from the solid-stemmed durum wheat cultivar CDC Fortitude (9) to disrupt the expression of *SSt1* and induce stem hollowness. Approximately 1.5 kg of seeds was soaked in tubs containing 0.5% (v/v) EMS solution for eight hours under gentle agitation, followed by four hours of continuous rinsing in fresh tap water. The seeds were dried overnight in a fume hood and sown in space-planted field plots near Saskatoon (SK), Canada, the following day. A single spike was harvested from each M1 plant, and the seeds were planted as 2,376 individual M2 head-rows the following field season. Ten M3 spikes were harvested from each row and saved for future use. Plants from each row were cut into cross-sections and rated for stem solidness, and any rows segregating for stem hollowness were noted. This led to the selection of the hollow-stemmed *pithless1* mutant.

Generating *TdDof* overexpression lines in the Kronos and *pithless1* backgrounds. Transgenic wheat lines overexpressing the *TdDof* gene were generated using the hollow-stemmed durum wheat cultivar Kronos and the *pithless1* mutant according the the procedures described in (10). The *TdDof* coding sequence was extracted from the Svevo annotation (7), synthesized, and cloned into the *pUC57-Kan* vector (Genewiz). The gene was then cloned into binary vector *pSc4ActR1R2* under the control of the rice *ACTIN* promoter (11). The final binary plasmid, *pEW398-TdDof*, was transformed into *Agrobacterium tumefaciens* strain AGL-1 and co-cultivated with immature wheat embryos at 23°C in the dark, according to published protocols (11) for three days. *In vitro* culture of the plant material was performed as described previously (12), with the exception that regeneration was carried out on medium containing 5mg/l zeatin. DNA isolated from regenerated plantlets was analyzed by qPCR to determine the copy number of the neomycin phosphotransferase II selectable marker gene relative to an internal control (Table S1). T0 transgenic plants carrying the T-DNA bearing *TdDof* were grown under controlled environmental conditions at at 22ºC day/17ºC night, with a 16-hour light/8-hour dark photoperiod at 450 µmol $m^{-2}s^{-1}$ an intensity of. Seeds were harvested at maturity, and the main stems were rated for solid-stemmed expression using a standard 1-5 scale (13). All lines were advanced to the T1 generation for further copy number validation, gene expression profiling, and microscopy analyses.

Chromium whole-genome sequencing. To examine structural variation around the *SSt1* interval, whole-genome sequencing was performed on solid-stemmed lines CDC Fortitude and W9262-260D3 and hollow-

stemmed lines Svevo, Kofa, and *pithless1* using the Chromium (10x Genomics) platform. Nuclei were isolated from ~30 seedlings per line as outlined in (14). High molecular-weight (HMW) genomic DNA was extracted from nuclei using a modified CTAB extraction protocol. Genomic DNA was quantified by fluorometry using Qubit 2.0 (Thermo Fisher), and size selection was performed to remove <40-kb fragments using pulsed field electrophoresis on a Blue Pippin instrument (Sage Science) according to the manufacturer's specifications. Final DNA integrity and size were determined using a TapeStation 2200 (Agilent) and Qubit 2.0 (Thermo Fisher) instrument, respectively.

Library preparation was performed as per the 10x Genome Library protocol (10x Genomics). For each sample, four uniquely barcoded libraries were prepared and multiplexed on the Illumina HiSeqX and HiSeq 2500 platforms. De-multiplexing was performed using the 10x Genomics software Supernova, and alignments to the Svevo reference genome (7) were performed using LongRanger software (10x Genomics). Structural variants were visualized using Loupe software (10x Genomics).

Whole genome and targeted Oxford Nanopore sequencing via CRISPR-Cas9 mediated enrichment of the *TdDof* region. HMW DNA was isolated from ~80 wheat seedlings of the solid-stemmed cultivar CDC Fortitude using a hybrid protocol that combines a modified version of the nuclei isolation method of (14), followed by the salting out method (10X Genomics) with minor modifications. Briefly, approximately 9 g of fresh leaf tissue was harvested from 2-week-old etiolated seedlings. The tissue was transferred to a chilled blender jar filled with 150 ml of ice-cold NIB buffer and pulse blended for 30s on low speed. The homogenate was filtered through two layers of cheesecloth and two layers of Miracloth into a cold beaker with H+20 buffer (14). The contents were mixed gently and incubated on ice for 45 minutes with occasional gentle mixing. The homogenate was pelleted at 3500g at 4°C for 30 minutes. The supernatant was carefully discarded, and the pellet was washed and re-pelleted three times in ice-cold homogenization buffer (14) (HB) at 4000 rpm at 4°C for 15 minutes. Following the final wash, the pellet was re-suspended in HB, divided into four Falcon tubes, and re-pelleted. The supernatant was discarded, and the pelleted nuclei were subjected to overnight lysis. Purified HMW DNA was precipitated by the addition of sodium chloride and absolute ethanol and retrieved by spooling with a glass rod. The DNA was washed in 70% (v/v) ethanol, air-dried, and re-suspended in elution buffer. HMW DNA was incubated at 4°C for re-hydration and quantified, size selected, and evaluated using Qubit 2.0, Blue Pippin, and TapeStation 2200 instruments as described above.

We performed two complementary library preparation protocols: CRISPR-Cas9 mediated enrichment of the *TdDof* region and standard whole-genome library preparation. For targeted sequencing of *TdDof*, we used the CRISPR-Cas9 mediated PCR-free enrichment protocol (Oxford Nanopore Technologies version: ENR_9084_v109_revD_04Dec2018) as a guideline for probe design. The boundary sequences flanking the *TdDof* region of interest (ROI) were extracted from the Svevo reference genome (7) and used for crRNA design. Candidate crRNA probes were identified within the boundary sequence using CRISPR RGEN Tools (15); these probes consisted of a 20-mer target sequence. The Svevo and RefSeq v1.0 wheat sequence (8) were used as reference sequences to evaluate the candidate probes for off-target sites and other efficiencies (i.e. self-complementarity). Two upstream and two downstream crRNAs were selected for use in targeted sequencing of the ROI. Library preparation followed the Cas-mediated PCR-free enrichment protocol (Oxford Nanopore Technologies version: ENR_9084_v109_revD_04Dec2018) using *Streptococcus pyogenes* Cas9 (Integrated DNA Technologies) for blunt end double-stranded cutting of the target DNA. To summarize, a fresh Cas9 ribonucleoprotein complex (RNP) was formed using a set of 1 or 2 crRNAs flanking the ROI. A pool of size-selected DNAs was dephosphorylated to protect non-target DNA from adapter ligation. The Cas9 RNP was combined with the dephosphorylated DNA sample, and sequencing adapters were ligated to cleaved and prepared DNA ends using an Oxford Nanopore 1D Ligation Sequencing kit (LSK109), followed by bead purification of long fragments. Approximately 392–1512 ng of the Cas9-enriched library was used as input across a total of 10 flow cells (Oxford Nanopore version R9.4.1 revD). Libraries for whole-genome Oxford Nanopore sequencing were also prepared using an Oxford Nanopore 1D Ligation Sequencing kit (LSK109) with HMW DNA that was purified and size selected as described above. Sequencing was performed on a GridION instrument using standard parameters and flow cells (R9.4.1 revD).

***De-novo* Assembly of the *TdDof* region in CDC Fortitude using Oxford Nanopore and 10x Genomics Chromium read data.** Oxford Nanopore read data were pre-processed with Porechop (https://github.com/rmcolq/Porechop) to remove adapters and NanoFilt (16) to remove reads with low

average base quality and reads <500 bp long. After pre-processing, the read length N50 was 5.6 kb. Reads from the targeted CRISPR-Cas9 were assembled using the miniasm assembler (17) to produce a preliminary assembly of the *SSt1* target region with an N50 of 23.7 Kb including a 103,345 bp contig containing three copies of the *TdDof* gene. To extend the assembly past the initial ROI, we supplemented the data with whole-genome Oxford Nanopore read data that aligned to the *SSt1* region in the Svevo pseudomolecule assembly (7). Alignment-based filtering was performed via two steps. First, minimap2 (18) was used to identify Oxford Nanopore reads that mapped to the *SSt1* region in Svevo. Second, LongRanger alignment software (10x Genomics) was used to align Chromium linked short reads to the Svevo assembly. Reads that mapped to the *SSt1* region were extracted, as were reads that did not map to *SSt1* but likely originated from the same molecules (shared GemCode tag). These reads were mapped to the pre-processed Oxford Nanopore whole-genome sequencing read data to identify additional long-read data that did not align well to the *SSt1* region in Svevo, effectively filling gaps that resulted from sequence dissimilarity between CDC Fortitude and Svevo. The extended long-read data were assembled using Canu (19) and merged with the draft miniasm assembly using ABACUS software to create the final assembly of the *TdDof* region.

RNA sequencing. A panel of durum wheat cultivars with varying levels of stem solidness was selected for transcriptomic analysis. We selected CDC Fortitude and W9262-260D3, which are solid-stemmed cultivars derived from the German cultivar Biodur. The hollow-stemmed cultivars included Kofa and Langdon (LDN) and the loss-of-function mutant line *pithless1*, which was selected from the CDC Fortitude mutant population. We also included the solid-stemmed line LDN-GB-3B; this solid-stemmed chromosome substitution line of Langdon carries chromosome 3B from Golden Ball, a hypothesized second source of stem solidness that exists in durum wheat. This experiment was performed in 3 replications per genotype. Three seeds per line were planted in 4 L pots and grown in a growth cabinet under T5 fluorescent lighting. Growth conditions were set to temperature cycles of 22°C during the day and 18°C at night, with a 16-hour photoperiod and light intensity of 600 µmol m^{-2} s^{-1}. Each pot was considered to be one treatment, and each treatment was grown in three replications. The experiment was arranged in a completely randomized design, and pots were randomly moved to a new position in the growth cabinet every seven days.
The main stems of three plants per treatment were sampled at Zadoks stage 32, when the first two nodes are present on the stem. Approximately 0.5 cm of the stem was sampled, measuring from the bottom of the lowermost node towards the uppermost node. The samples were immediately placed in 1.5 mL micro-centrifuge tubes, flash-frozen in liquid nitrogen, and stored at −80°C prior to RNA extraction. Stem tissue was ground in liquid nitrogen with a sterilized mortar and pestle. Total RNA extraction was performed using a Qiagen RNeasy Plant Mini Kit (Qiagen) as per the manufacturer's protocol. RNA integrity was evaluated using an Agilent Bioanalyzer RNA 6000, and RNA quantification was performed using a Qubit 2.0 Broad Range assay kit (Thermo Fisher).
Individually barcoded cDNA libraries were prepared using a TruSeq RNA v2 unstranded kit (Illumina) as per the manufacturer's protocol. Library integrity was assessed on an Agilent Bioanalyzer using a high sensitivity DNA analysis kit. Library quantitation was performed using a Qubit 2.0 High Sensitivity assay kit. Individually barcoded libraries were diluted to 10 ng μl^{-1}, pooled into groups of six, and sequenced across five lanes on the Illumina HiSeq4000 platform with 2 x 150 bp PE chemistry.

RNAseq bioinformatics analysis pipeline. We used standard bioinformatics procedures to process that RNAseq data. Adaptor and quality trimming was performed using Trimmomatic version 0.27 (3) with the parameters ILLUMINACLIP:TruSeq3-PE:2:30:10 LEADING:3 TRAILING:3 SLIDINGWINDOW:4:20 MINLEN:75. Trimmed reads were assessed for adaptor contamination and quality using FastQC and aligned to the Svevo reference sequence (7) using STAR version 2.5 (20) with default parameters, except that the maximum mismatch rate (--outFilterMismatchNmax) was set to 6 (minimum 96% sequence identity) and the maximum intron length (--alignIntronMax) was set to 10,000 bp. Alignment files containing aligned reads were input into StringTie (21) to count reads mapping to genes in the Svevo annotations. A matrix of raw read counts was normalized by DESeq2 (21) to identify differentially expressed genes between hollow and solid-stemmed lines. Pairwise comparisons were made between the three hollow and five solid-stemmed lines (Table 5.1). The significance threshold for declaring a gene to be differentially expressed was defined as an adjusted $p < 0.01$.

Weighted gene co-expression network analysis. Unsigned RNAseq co-expression network analysis was performed using the WGCNA (22) R package (default parameters). The network was constructed from count estimates for 36,403 genes (filtered to remove genes expressed at low levels, i.e., mean raw count > 15) across all 21 samples, combined with stem solidness data across all samples that were rated at maturity (1 to 5 scale). In total, 37 co-expression modules were detected. From this global network, a co-expression sub-network was extracted in two steps. First, filters were applied to pairwise (hollow- vs solid stemmed) gene expression data to extract a final list of 12 genes that met the criteria of being differentially expressed (adjusted $p < 0.05$) across all pairwise comparisons (Table 1). Next additional genes were extracted from the global network if their expression showed high correlation ($-0.94 < r > 0.94$) with at least one of these 12 genes. The resulting subnetwork consisted of 32 genes representing 7 out of 37 total co-expression modules.

Stem fixation for light and electron microscopy. Fresh hand-cut stem sections of CDC Fortitude, *pithless1*, Kofa, and T1 transgenic lines of *pithless1* were taken approximately 2 cm above the node and submerged in 25 mM piperazine-N-N' bis (2-ethanolsulfonic acid) (PIPES), pH 7.0 containing 2% (v/v) glutaraldehyde in glass scintillation vials. Following overnight incubation at 4°C, the samples were washed with 25 mM PIPES and fixed in 2% OsO_4 in 25 mM PIPES at room temperature for 1.5 hours. The fixed samples were washed in 25 mM PIPES and dehydrated in an ascending graded ethanol series (30%, 50%, 70%, 95%, and three 100% exchanges). The subsequent sample preparation steps for scanning electron microscopy (SEM) differed from those used for light microscopy and transmission electron microscopy (TEM), as detailed below.

For SEM, after sample dehydration, the ethanol was substituted with amyl acetate using increasing ratios of amyl acetate to ethanol (spanning 1:3 parts, 1:1, 3:1, then two pure amyl acetate exchanges). All solvent exchanges were separated by 15 minutes. The samples were critical-point dried with solvent-substituted liquid CO_2 (Polaron E3000 Series II), mounted on aluminum specimen stubs with conductive carbon glue (Ted Pella, Inc.), and rotary coated with 10 nm of gold (Edwards S150B sputter coater). Imaging was performed with a 3 kV accelerating voltage, 10 uA current, and working distance of 3.8 mm on a Field Emission SEM (Hitachi SU8010).

For sample preparation for light microscopy and TEM, samples in 100% ethanol were transferred to ice cold propylene oxide (dropwise until the propylene oxide: ethanol ratio was approximately 2:1). After 30 minutes, two changes with cold propylene oxide were performed, separated by 30 minutes. To embed the samples, sample changes were done with a series of propylene oxide: Spurr’s resin (23) mixtures, moving from 2:1, 1:1, 1:2 every 30 minutes, then adding 100% Spurr’s overnight. After a final exchange in fresh Spurr’s resin, the samples were positioned in polyethylene embedding capsules (Electron Microscopy Sciences) and heated for 24–48 hours at 60°C. Sectioning was performed on a Reichert Ultracut E microtome. For light microscopy, semi-thin sections were stained with Toluidine Blue (1% in 1% Borate) and imaged under a Leica DMR microscope with an Optronics MacroFire Color camera. For TEM imaging, fine details of ultrastructure were examined with the acceleration voltage of 120 kV on a high contrast/resolution digital transmission electron microscope (Hitachi HT7700).

***In situ* PCR of *TdDof*.** *TdDof* transcript localization in paraformaldehyde-fixed paraffin-embedded internode cross-sections was performed by *in situ* PCR. Sample and reagent preparation were carried out based on the combination of protocols described by (24, 25) with some modifications and additional details outlined below. Zadoks stage 32 and stage 34 internode samples from *pithless1*, Kofa, and CDC Fortitude were fixed in fresh 4% paraformaldehyde in phosphate buffered saline (PBS). After washing and dehydration, the samples were embedded in paraffin and sectioned to 10 μM using a histology microtome. Subsequent treatments including dewaxing, rehydration, Proteinase K treatment, *in situ* reverse transcription, *in situ* PCR, and staining were performed on slides in Frame-Seal incubation chambers. Reverse transcription oligonucleotides (DG036, 5′-ATTGCCGAAGCTGATTGAG-3′) and PCR primers (DG015, 5′-CAACCAGCTTCAGCTGCAG-3′; DG038, 5′-TGACCATGATTGTTGTTGTTTCC-3′) were designed to specifically target *TdDof* to exclude off-target effects from *TdDof* homologous and homeologous genes. Intron-spanning features were also included in the primer design to eliminate potential genomic DNA contamination. *In situ* reverse-transcription of *TdDof* mRNA was carried out on DNase-treated internode sections using the SuperScript IV VILO system (ThermoFisher). This step was omitted in the

corresponding negative control slides. PCR was carried out using Phusion High-Fidelity DNA Polymerase (Invitrogen) containing 4 µM (final concentration) of Digoxigenin-11-dUTP (DIG) (Sigma) as an additional reagent. Colorimetric detection of DIG-labeled PCR products was performed with anti-DIG-AP (Sigma), and the sections were stained with BM-purple (Sigma). Visualization was processed using a Leica DMR equipped with a MicroFire camera (Optronics) under bright field Illumination.

Viability staining. Fresh hand-cut stem cross-sections of CDC Fortitude and *pithless1* taken approximately 2 cm above the second node were submerged in PBS prior to staining. Stock solutions of the stains propidium iodide (PI) and fluorescein diacetate (FDA) were prepared at 20 µg mL^{-1} for PI and 2 µg mL^{-1} for FDA. Each stem section was stained with PI for 10 minutes, followed by PI and FDA for 10 minutes. After staining, the stem sections were placed on a glass slide in PBS beneath a 1.5 mm coverslip and imaged immediately under a confocal microscope (Zeiss LSM510 with motorized Axiovert 200 microscope) equipped with a 25X water immersion objective lens (Plan-Neofluar 25x/0.8 1mm corr Ph2) and ConfoCor2 v3.2 software. For PI visualization (Channel 3), excitation at 543 nm and BP 585–615 nm filter was used. For FDA visualization (Channel 2), 488 nm excitation and a BP 505–530 nm filter was used. Single XY plane scans are presented. The multi-track imaging software feature enabled both excitation wavelengths to be used in succession with a detector gain of 583 and pinhole of 252 for Channel 2 and a gain of 1250 and pinhole of 233 for Channel 3.

Terminal deoxynucleotidyl transferase (TUNEL) assay. Zadoks 34 stage internode samples from *pithless1* and CDC Fortitude were collected and fixed in fresh 4% paraformaldehyde in phosphate buffered saline (PBS), pH 7.5. Following overnight incubation at 4°C, the samples were washed with PBS and dehydrated in an automated ascending grade ethanol series (3 x 70%, 80%, 95% for one hour each, followed by three 100% exchanges for two hours each), followed by xylene exchanges (two exchanges incubated for two hours each) and paraffin wax exchanges at 62°C for 2 hours each. Samples were cross-sectioned to 10-20 µm using a rotary microtome (Leitz 1212). Subsequent treatments including dewaxing, rehydration, and proteinase K treatment were conducted on slides. TUNEL assays were performed using the TACS® 2 TdT Fluorescein Kit (Trevigen, catalog number 4812-30-K) following the provided protocol, with a 30-minute Proteinase K treatment and cadmium cation in the labeling reaction mix. An unlabeled experimental control, in which TdT enzyme was omitted, and a positive control, in which TACS nuclease treatment was used, were performed alongside each assay. Selected sections were mounted with Antifade Mounting Medium with DAPI (VECTASHIELD, H-1200-10) for DAPI staining and sealed with permount and a coverslip. Imaging was performed by confocal laser scanning microscopy with 405 nm excitation and BP 420-450nm for DAPI and 488 nm excitation and BP 505-530nm for fluorescein visualization (Zeiss LSM 880). Single XY planes are presented in Figure 5. Z stack projections for experimental treatments captured 10 XY plane slices with a 2 µm step size, and unlabelled and positive controls covered 7 slices separated by 1 µm steps, as presented in Supplementary Figure S5.

Metabolite analysis. Tissue was sampled from stem tissue of Kofa, CDC Fortitude, and *pithless1* plants at Zadoks stage 32, 33, and 34. Three biological replicates per sample were further processed and analyzed by Metabolon, Inc. (www.metabolon.com) for global unbiased metabolite profiling involving a combination of three platforms: ultra-HPLC–tandem mass spectrometry optimized for basic species, ultra-HPLC/tandem mass spectrometry optimized for acidic species, and gas chromatography–mass spectrometry. The methods used were described previously **(26, 27)**. For normalization, the samples were scaled to the median value for each compound, and two-way ANOVA was performed among different replicates for analysis of variance. For statistical significance testing, *p*-values were calculated and sorted to interpret the q-values, which were further used to estimate the false discovery rate of the selected compound list.

Confirmation of copy number variation and gene expression data using ddPCR. Copy number and gene expression of *TdDof* were validated using the droplet digital PCR (ddPCR) system (Bio-Rad). Probe primers (D1 and D2) were designed specifically to exclude off-targets from their homologous and homoeologous genes. A pair of primers (B1) was designed to target the unique boundary sequence. A single copy marker close to *TdDof* on chromosome 3B was used as a reference for copy number variation

(CNV) analysis, and the housekeeping gene *TaActin* was used as a reference for gene expression analysis. All primers contained at least one SNP compared to homoeologs in the other chromosome. Primer sequences for each target and reference genes are provided in Supplementary Table S1.
High-quality DNA was extracted using the CTAB method and diluted to a final concentration of 1–5 ng μL^{-1} for the reaction. EcoRI and HindIII (NEB) digestions were performed prior to the ddPCR assay. The extracted RNA (using the methods described above) was treated with DNase I (Thermo Fisher Scientific), and reverse transcribed using the SuperScript IV VILO system (Thermo Fisher Scientific) according to the manufacturer's instructions. CNV and transcript abundance were measured using the Bio-Rad QX200 ddPCR System (Bio-Rad). In brief, each 20 µL 1 x ddPCR EvaGreen SuperMix containing DNA/cDNA templates, forward and reverse primers at optimized concentrations was mixed with 70 µL of Droplet Generation oil for EvaGreen (Bio-Rad) in a DG8 Cartridge (Bio-Rad). The cartridge was covered with a DG8 gasket and loaded into the QX200 Droplet Generator (Bio-Rad) to generate PCR droplets. 40 µL of each droplet mixture was transferred to a 96-well PCR plate (Bio-Rad) and sealed using a PX1™ PCR plate Sealer (Bio-Rad). PCR thermal cycling conditions were optimized, and the amplification signals were read using a QX200™ Droplet Reader and analyzed using QuantaSoft software (Bio-Rad).

Marker development for breeding. To develop a high-throughput KASP marker for breeding, we mined our database of Chromium sequence data to call SNPs and design additional markers. This approach has several benefits over exome-based strategies for marker development, as it includes the presence of read data in intergenic regions, higher per-base read coverage, and the ability to more accurately resolve repetitive and complex genomic regions with long range data, which spanned an average of 40 kb when aligned to the Svevo reference sequence (see 'Chromium whole-genome sequencing'). Alignments were used for variant calling with FreeBayes, and only homozygous variants were retained. The retained SNPs were converted into KASP markers using Primer3 software. Finally, *in silico* validation of specificity was performed by mapping the primer sequences to the Svevo reference sequence by BLASTn, and only uniquely mapping primer pairs were retained and validated using a diversity panel consisting of hollow and solid-stemmed durum and bread wheat cultivars. This method was successfully used to select a new marker (*usw275*) (Fig. 1D, Table S1, Fig. S7) that maps within the fine-mapped interval (Svevo chromosome 3B position: 829,114,574) of *SSt1* and scores hexaploids and tetraploids correctly.

Supplementary Figures

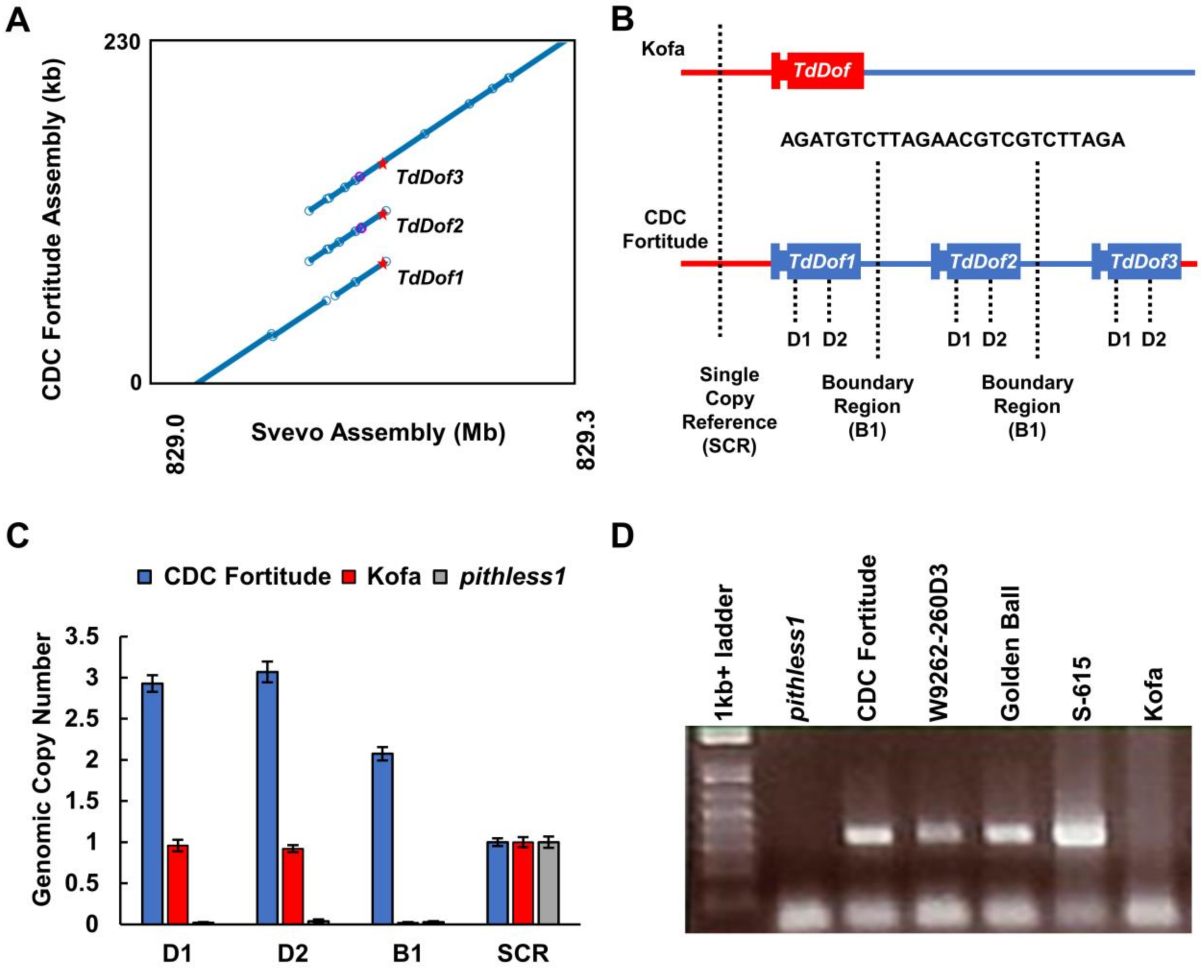

Figure S1. Diagram showing the organization of the *TdDof* CNV region in solid-stemmed cultivars. **A)** Alignment of the hybrid assembly containing three copies of *TdDof* in CDC Fortitude (Y-axis) to the Svevo reference genome (X-axis). The three *TdDof* copies are numbered from $TdDof_1$-$TdDof_3$ beginning with the most proximal copy (red stars) Three copies of the *TdDof* gene (*TdDof1-3*) are present in the genome of solid-stemmed wheat (CDC Fortitude) compared to the single copy in hollow-stemmed wheat (Kofa). **(B)** At the CNV breakpoint between each *TdDof* copy in the solid-stemmed cultivars, a unique 25 bp insertion occurs at the breakpoint between *TdDof1-2* and *TdDof2-3* (B1). The 5′ upstream regions of *TdDof* (Kofa) and *TdDof1* (CDC Fortitude) are identical (red segment). Similarly, the 3′ downstream region of *TdDof* is identical to the downstream region of *TdDof3* in CDC Fortitude. **(C)** ddPCR primers were designed to validate our model at the 5′ upstream region (SCR), the boundary sequence insertion (B1), and the internal *TdDof* gene (D1, D2). These results confirm that CDC Fortitude carries three copies of the *TdDof* gene compared to a single copy in Kofa and no copies in the *pithless1* mutant (primer sets D1, D2). Two copies of the boundary sequence were present in CDC Fortitude compared to no copies in either Kofa of *pithless1* (primer set B1). One copy of the upstream region was present in all three lines (primer set SCR). **(D)** Agarose gel image of the boundary region insertion (B1) sequence marker *TdDof_CNV* screened in different sources of stem solidness. Amplification of PCR product indicates the presence of multiple *TdDof* copies, as observed for solid-stemmed wheat lines CDC Fortitude and W9262-260D3 (derived Biodur), Golden Ball, and S-615. The lack of PCR product amplification was observed for hollow-stemmed lines *pithless1* and Kofa, consistent with the deletion of this region in *pithless1*, and the lack of *TdDof* CNV in Kofa.

TRITD3Bv1G280530 (TdDof) Nucleotide Sequence (1134 bp)

ATGATCTTCCCTCCTGCCTTCCTCGACTCATCAAGCTGCTGGAACACCAACCACAACCAG ← Exon1
CTTCAGCTGCAGCAAATCGGCAGTAACACTCATATCACTACTACTCCTTCACCTGCTGGC
CATGGTCCTGGAGACGGAGGAGGCGGAAACAACAACAATCATGGTCAGCAGGAAGGATTA
ATGGCCACGGCCGGGGCGGGAGGAGGTGGTGGTGATGGTGGTGGCGGCGGCGGTGGGGAT
GGTGACAGCGCCAGCGGCGGGAACAACAAGCCGATGTCGATGTCGGAGCGGGCGCGGCTG
GCGCGGGTGCCACAGCCGGAGCCGGGGCTCAACTGCCCGCGCTGCGATTCCACCAACACC
AAGTTCTGCTACTTCAACAACTACTCCCTCACCCAGCCCCGCCACTTCTGCCGGGCCTGC
CGCCGCTACTGGACCCGCGGCGGCGCGCTCCGCAACGTCCCCGTCGGCGGAGGGTACCGT ← Exon2
CGCCACGCCAAGCGCAGCACCAAGCCCAAGGCCGGGTCGGCTGGATCCGGAACTGCCGCG
GCAGGGACGTCGTCTGCGACGTCGACGACGCCCAGCACCACTGCTTGCACCACCGGCACA
GCTGCCACTGCGCCGCCCGCTCTGCAGTACTCCATGTTCGGCAGCGCGCCGCCGCACAGC
AGCCGGTTCGCCGATAGCTTCGACCCCGCGAGCCTCGGCCTCAGCTTCCCCGCCAGGCTG
CTCTTCCCCGACAATGGCGCCTACGCTGCCGACGGTGGCGCGCAGCAGCACCACCACCAC
CAGGGGAACGGGAACGGCATGGAGCAGTGGGCGGCTGCGCACATGCAGAGCTTCCCGTTC
CTGCACGCCATGGACCACCAGATGTCCGGGAATCCTCAATCAGCTTCGGCAATGCCAACC
ACAATGGCGGCGATGCAGGGCATGTTCCACCTCGGGCTACAGAGCGGCGGCGGCGGCGGT
AATGGCGACGATGGGGGAAACCACCAGTTCCACCACCAGCCGGCCAAGAGGGACTACAAC
CAGCAGCAGCAGCAGGATTACCCAAGCAGCAGGGGCATGTACGGGGACGTGGTCAATGGC
AATGGCGGCGGCTTCAATTTCTATTCCAGCACTAGCAATGCAGCTGGTAATTAG

TRITD3Bv1G280530 (TdDof) Protein Sequence (377 aa)

MIFPPAFLDSSSCWNTNHNQLQLQQIGSNTHITTTPSPAGHGPGDGGGNNNNHGQQEGL
MATAGAGGGGGDGGGGGGGDGDSASGGNNKPMSMSERARLARVPQPEPGLNCPRCDSTNT
KFCYFNNYSLTQPRHFCRACRRYWTRGGALRNVPVGGGYRRHA**KRSTKPKAGSAGSGTAA** ← zf-Dof Domain
AGTSSATSTTPSTTACTTGTAATAPPALQYSMFGSAPPHSSRFADSFDPASLGLSFPARL
LFPDNGAYAADGGAQQHHHHQGNGNGMEQWAAAHMQSFPFLHAMDHQMSGNPQSASAMPT
TMAAMQGMFHLGLQSGGGGGNGDDGGNHQFHHQPAKRDYNQQQQQDYPSSRGMYGDVVNG
NGGGFNFYSSTSNAAGN*

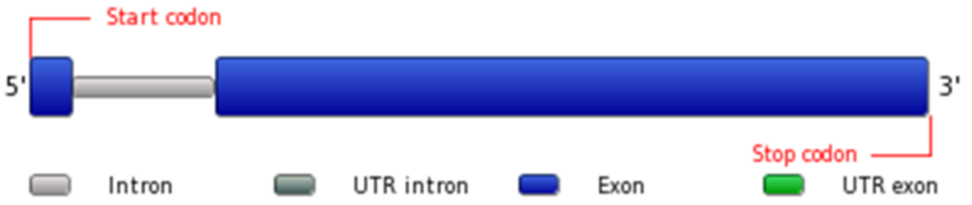

Figure S2. Nucleotide and protein sequences of the *TdDof* gene (*TRITD3Bv1G280530*), encoding a putative Dof (DNA-binding with one finger) protein. The zf-dof DNA binding domain is indicated in red font.

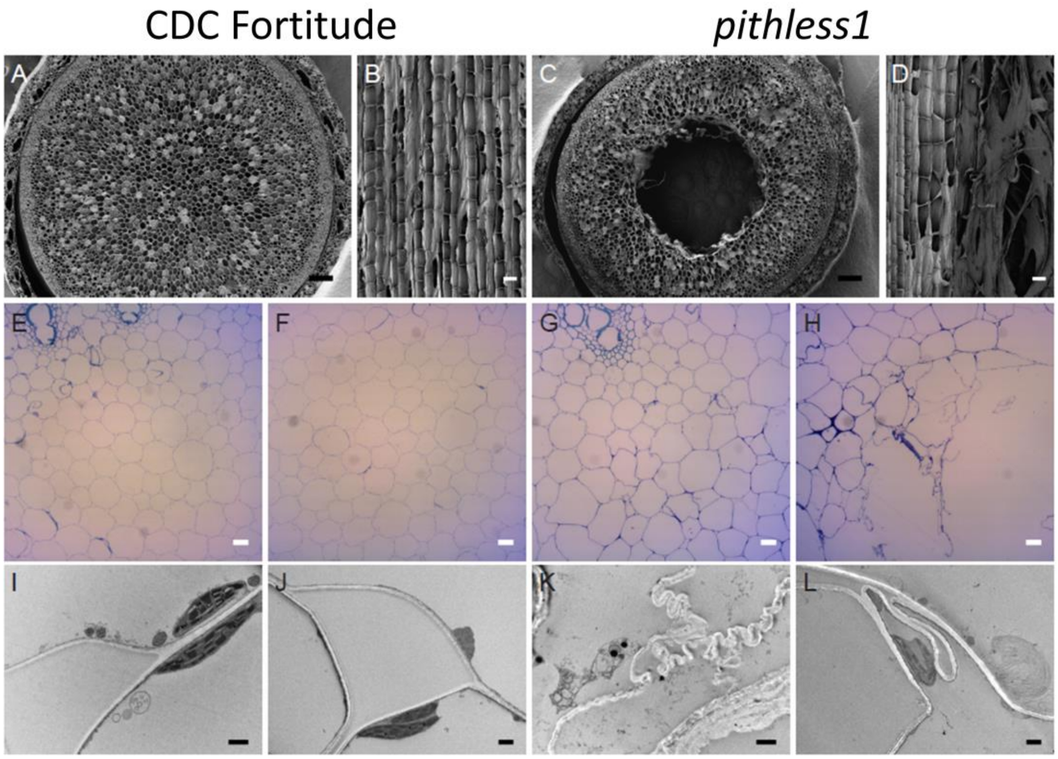

Figure S3. Stem pith anatomy in CDC Fortitude and mutant *pithless1* plants sampled at Zadoks stage 32. The stems exhibited a solid pith in CDC Fortitude and a hollow pith in *pithless1* when viewed by scanning electron microscopy (A–D), light microscopy (E–H) and transmission electron microscopy (I–L) in cross-sectional planes (A, C, E–L) and longitudinal planes (B, D). In CDC Fortitude, parenchyma cells in and surrounding the central pith region appeared intact and approximately uniform in size (E, F) with even, continuous cell walls (I) and triangular three-way junctions between cells (J). In contrast, in *pithless1*, parenchyma cells surrounding the central pith region appeared irregular and angular (G) or broken and collapsed (H), with undulating cell walls (K) and three-way junction buckling (L). Bars = 250 µm (A, C), 50 µm (B, D), 20 µm (E–H), 1 µm (I–L).

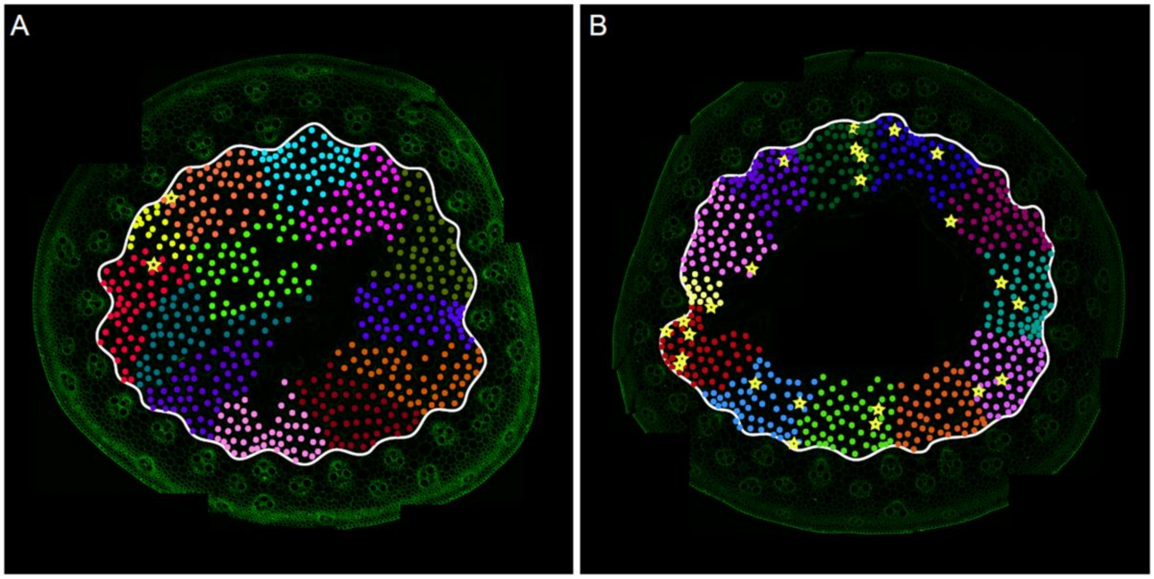

Figure S4. Quantifying cell death signals in CDC Fortitude and *pithless1* using TUNEL. Stem cross-sections for **(A)** CDC Fortitude and **(B)** *pithless1* were visualized through a series of stitched confocal laser-scanning microscopy images. Intact cells within the central pith zone (outlined in white) were counted using colored dots to mark every 50 cells, with yellow dots marking the remaining cells. Fluorescent nuclei (indicating cell death signals) are marked by yellow stars.

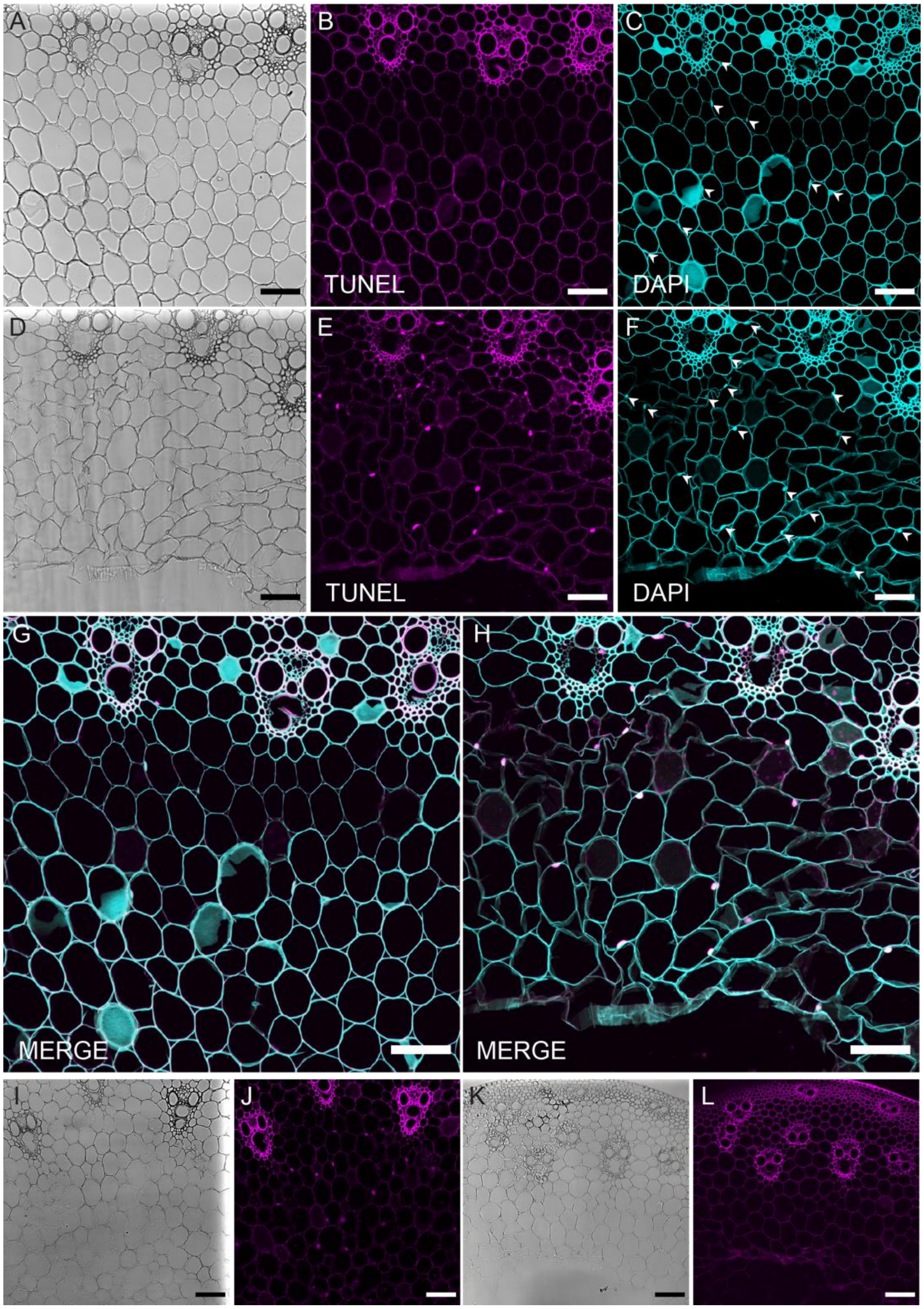

Fig. S5. TUNEL assay controls. Co-localization of DAPI nuclear counterstain with nuclear label from a TUNEL assay for DNA fragmentation, in representative CDC Fortitude (**A-C, G, I, J**) and mutant *pithless1* (**D-F, H, K, L**) stem cross-sections, visualized with confocal laser scanning microscopy; **A, D, I, K:** phase-contrast image; **B, E, J, L:**TUNEL assay fluorescein signal; **C, F:** DAPI signal: and **G, H:** TUNEL assay fluorescein-DAPI merge. DAPI-positive nuclei are marked by white arrowheads. Representative TUNEL assay positive control shown in **I and J;** and K and L represent unlabeled control stem cross-sections, using TACS nuclease treatment of *pithless1* stem cross-section and TdT enzyme omission for CDC Fortitude stem cross-section, respectively. Images in A-H and I-L are Z-stack projections of the same thickness and step interval (as detailed in methods). Bars = 100 µm.

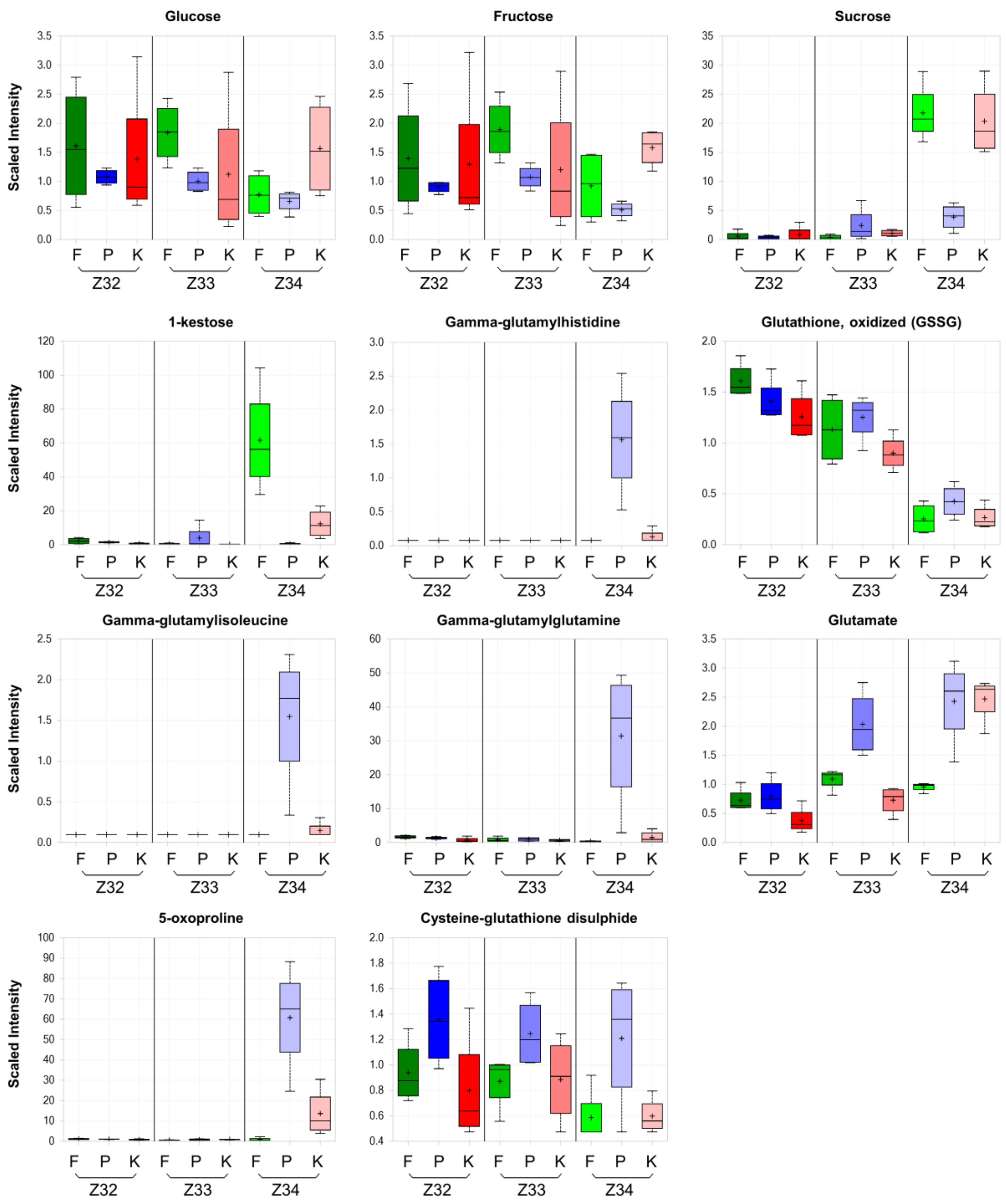

Fig. S6. Metabolite analysis of stem tissue collected from CDC Fortitude (F), *pithless1* (P), and Kofa (K) at three different time points. Zadoks stage 32 (Z32), Zadoks stage 33 (Z33), and Zadoks stage 34 (Z34). Three biological replicates per sample were analyzed for global unbiased metabolite profiling involving a combination of three platforms: ultra-HPLC–tandem mass spectrometry optimized for basic species, ultra-HPLC/tandem mass spectrometry optimized for acidic species, and gas chromatography–mass spectrometry. The Y axis of each plot represents the scaled intensity across all samples (median value across all samples = 1) for each biomolecule. The horizontal line within the body of the boxplot denotes the median value, whereas the '+' symbol denotes the mean. The upper and lower quartiles are denoted by the upper and lower bounds of each box. The whiskers represent the most extreme upper and lower value in each dataset.

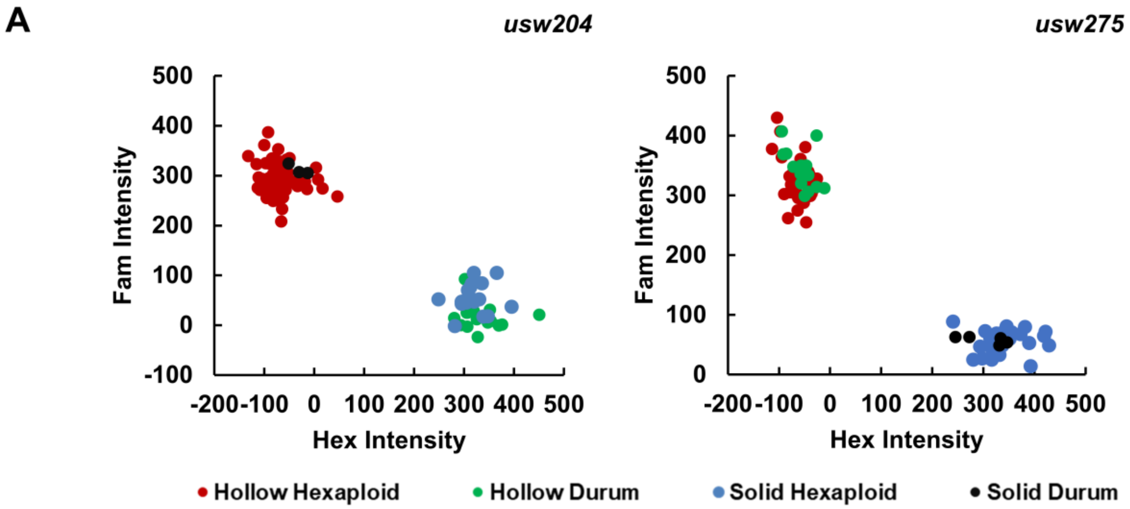

B

Svevo Chromosome 3B Position		Alternate Allele	CDC Fortitude	*pithless1*	W9262-260D3	Kofa	Svevo	CDC Landmark	CDC Stanley	
827,464,009	G	A	1	1	1	0	0	0	1	
827,903,953	T	C	1	1	1	0	0	0	1	
827,907,191	C	T	1	1	1	0	0	0	1	
827,924,063	G	C	1	1	1	0	0	0	1	
828,949,943	G	A	1	.	1	0	0	1	0	
829,114,574	C	T	1	.	1	0	0	1	0	* *usw275*
829,629,899	C	T	1	1	1	0	0	1	0	
829,871,761	A	G	1	1	1	0	0	0	1	
829,915,344	T	G	1	1	1	0	0	0	1	
830,609,000	A	G	1	1	1	0	0	0	1	* *usw204*
834,437,859	T	C	1	1	1	0	0	0	1	

Figure S7: Developing markers for high-throughput selection of stem solidness for use in breeding programs. **A)** Comparison of the previously reported KASP marker *usw204* (13) and newly developed KASP marker *usw275*. The previously reported marker *usw204,* although diagnostic for stem solidness, scored durum and bread wheat inversely. The new marker *usw275* scores both wheat species correctly and is therefore more suitable for high-throughput marker-assisted selection. **B)** Allelic phase shift between solid-stemmed durum (CDC Fortitude and W9262-260D3) and bread wheat (CDC Landmark) cultivars affects marker scoring within *SSt1*. Marker haplotypes around the *SSt1* region are shown, where '1' (red) indicates the solid allele and '0' (blue) indicates the hollow allele relative to the scoring in durum wheat, and "." indicates a lack of amplification. An allelic phase shift was identified within Svevo positions 828.5 and 829.6 Mb consistent with the *SSt1* fine mapping interval including the marker *usw275*, where hexaploids and tetraploids were scored correctly.

Supplementary Tables

Table S1. Primers used in this study

Marker Name	Sequence (5′ to 3′)	Experiment
usw305HF	AGACATACCATCGCTTCATCGG	Fine mapping
usw305FF	AGACATACCATCGCTTCATCGA	Fine mapping
usw305R	ATCCTTTTCTTCCTCACCTTCC	Fine mapping
usw306HF	TACGCATGCATGTGGTTGTTTAT	Fine mapping
usw306FF	TACGCATGCATGTGGTTGTTTAG	Fine mapping
usw306R	CTTTGCATCGATTACTGTTGGT	Fine mapping
usw307HF	TTTTGGTTTTCCGTGATTAACTAA	Fine mapping
usw307FF	TTTTGGTTTTCCGTGATTAACTAG	Fine mapping
usw307R	GCAAATAAATTCAGACACCAAGC	Fine mapping
usw308HF	AGACTATGCTTGGCGATTCGTA	Fine mapping
usw308FF	AGACTATGCTTGGCGATTCGTG	Fine mapping
usw308R	AAGGTCAAACCTTGGATTTTCA	Fine mapping
ek08_5169F	AAGCATGGGATGAGAGGAGATA	Fine mapping
ek08_5169R	GCCATAGAGAATGCTCCTGTTC	Fine mapping
gwm247F	GCAATCTTTTTTCTGACCACG	Fine mapping
gwm247R	ATGTGCATGTCGGACGC	Fine mapping
usw275HF	GAAAACAAAACCTGTCAAAAAT	Marker-assisted selection
usw275FF	GAAAACAAAACCTGTCAAAAAC	Marker-assisted selection
usw275R	GAATTTTCGGAGTTACAGATTGC	Marker-assisted selection
usw204FF	AGACTATGCTTGGCGATTCGTA	Marker-assisted selection
usw204FF	AGACTATGCTTGGCGATTCGTG	Marker-assisted selection
usw204R	AAGGTCAAACCTTGGATTTTCA	Marker-assisted selection
TdDof_CNV_F	AGATGTCTTAGAACGTCGTCTTAGA	*TdDof* insertion sequence
TdDof_CNV_R	TCTAAGACGACGTTCTAAGACATCT	*TdDof* insertion sequence
GamyB1F	GATCCGAATAGCTGGCTCAAGTAT	Transgenic plant validation
GamyB2R	GGAGACTGCAGGTAGGGATCAAC	Transgenic plant validation
GamyB1P	JOE-CGTGGCTCCTGCGATGCAGC-TAMRA	Transgenic plant validation
Npt2B2F	CTCCTGCCGAGAAAGTATCCA	Transgenic plant validation
Npt2B4R	GCCGGATCAAGCGTATGC	Transgenic plant validation
Npt2B2P	FAM-TGGCTGATGCAATGCGGCG-TAMRA	Transgenic plant validation
D1_F	GTTCCTGCACGCCATGGAC	*TdDof* CNV region validation
D1_R	TCCCCCATCGTCGCCATTA	*TdDof* CNV region validation
D2_F	GATGTCCGGGAATCCTCAAT	*TdDof* CNV region validation
D2_R	TAGTCCCTCTTGGCCGGCT	*TdDof* CNV region validation
B1_F	AGCCTTAGATGTCTTAGAACGTCG	*TdDof* CNV region validation
B1_R	CACATAGGTGAACTAGGACGTGTG	*TdDof* CNV region validation
Reference_F	TCGCAACGGCTACCAGAAG	*TdDof* CNV region validation
Reference_R	GGGCGGGCTCTGCTTTTA	*TdDof* CNV region validation

Table S2. High-confidence genes within the *SSt1* fine mapping interval on chromosome 3B in the Svevo gene annotations. Fine Mapping Interval on Chromosome 3B in the Svevo Gene Annotations. Genes in the gray shaded box were deleted in *pithless1*, including *TdDof* (highlighted in bold text).

Start Position	End Position	Gene Accession	Annotation
828,623,114	828,624,236	*TRITD3Bv1G280250*	Werner Syndrome-like exonuclease
828,686,787	828,687,872	*TRITD3Bv1G280300*	Disease resistance protein RPM1
828,700,609	828,704,982	*TRITD3Bv1G280310*	Vacuolar protein sorting protein 25
828,709,496	828,709,836	*TRITD3Bv1G280340*	Metallothionein
828,736,444	828,736,778	*TRITD3Bv1G280350*	Metallothionein
828,743,910	828,744,254	*TRITD3Bv1G280370*	Plant invertase/pectin methylesterase inhibitor superfamily protein G
828,745,475	828,747,104	*TRITD3Bv1G280380*	30S ribosomal protein S17
828,759,613	828,760,296	*TRITD3Bv1G280390*	30S ribosomal protein S19
828,833,595	828,837,436	*TRITD3Bv1G280440*	SANT domain-containing protein 2 G
828,867,581	828,868,220	*TRITD3Bv1G280460*	Very-long-chain (3R)-3-hydroxyacyl-CoA dehydratase
829,151,607	829,152,953	*TRITD3Bv1G280530*	**Dof zinc finger protein (*TdDof*)**
829,287,250	829,290,284	*TRITD3Bv1G280580*	Dof zinc finger protein
829,480,070	829,480,539	*TRITD3Bv1G280680*	transmembrane protein
829,481,310	829,489,485	*TRITD3Bv1G280690*	Cytochrome P450
829,484,951	829,485,802	*TRITD3Bv1G280700*	Ubiquitin-like modifier-activating enzyme 1 G
829,634,202	829,635,675	*TRITD3Bv1G280790*	Ankyrin repeat family protein
829,813,644	829,815,565	*TRITD3Bv1G280880*	Cytochrome P450
829,841,283	829,842,134	*TRITD3Bv1G280910*	Pre-mRNA-splicing factor ISY1-like protein
829,884,880	829,887,768	*TRITD3Bv1G280930*	Protein CHUP1, chloroplastic G
829,976,659	829,978,833	*TRITD3Bv1G280950*	Pectin acetylesterase
830,007,849	830,010,374	*TRITD3Bv1G280970*	Pectin acetylesterase
830,012,824	830,013,099	*TRITD3Bv1G280980*	Pectin acetylesterase
830,021,757	830,029,463	*TRITD3Bv1G280990*	Pectin acetylesterase
830,036,668	830,037,318	*TRITD3Bv1G281000*	Pectin acetylesterase
830,043,027	830,043,763	*TRITD3Bv1G281010*	Pectin acetylesterase
830,046,051	830,047,563	*TRITD3Bv1G281020*	91A protein G
830,065,376	830,067,734	*TRITD3Bv1G281040*	Pectin acetylesterase
830,164,390	830,165,032	*TRITD3Bv1G281080*	Histone H2A
830,165,442	830,166,543	*TRITD3Bv1G281090*	RNA-binding protein
830,249,836	830,251,657	*TRITD3Bv1G281140*	Receptor-like protein kinase
830,273,520	830,275,736	*TRITD3Bv1G281170*	DNA topoisomerase 3
830,293,567	830,294,027	*TRITD3Bv1G281180*	Histone H2A
830,296,115	830,296,444	*TRITD3Bv1G281190*	Outer-membrane lipoprotein LolB G
830,301,129	830,302,409	*TRITD3Bv1G281200*	alpha/beta-Hydrolases superfamily protein
830,323,385	830,323,996	*TRITD3Bv1G281220*	Inorganic pyrophosphatase protein
830,333,138	830,336,293	*TRITD3Bv1G281240*	Kelch repeat-containing F-box family protein
830,337,937	830,338,846	*TRITD3Bv1G281250*	Soluble inorganic pyrophosphatase
830,397,899	830,398,141	*TRITD3Bv1G281280*	Ubiquitin family protein
830,434,190	830,435,808	*TRITD3Bv1G281310*	Ethylene-responsive transcription factor, putative G

830,439,857	830,440,258	*TRITD3Bv1G281330*	Caffeic acid O-methyltransferase
830,551,081	830,558,494	*TRITD3Bv1G281340*	Protein upstream of flc
830,589,167	830,590,198	*TRITD3Bv1G281370*	MYB transcription factor

Table S3. Analysis of stem solidness and *nptII* copy number in *pithless1* and Kronos transformations with the *TdDof* overexpression construct. The hollow-stemmed mutant line *pithless1* and durum wheat cultivar Kronos were transformed using the *pEW398-sst1* construct, containing *TdDof* under the control of the rice *ACTIN* promoter and the nptII selectable marker gene. Non-transformed controls for each experiment were regenerated from calli without prior co-cultivation with Agrobacterium. Genomic copy number of the *nptII* are presented for each independent T0 line, along with the average whole stem solidness rating for each line (1 to 5 visual rating scale with 1 = very hollow-stemmed, and 5 = very solid-stemmed).

T0 Plants Transformed with *pEW398-sst1*	*nptII Copy Number*	Stem Solidness
*pithless1_pEW398-sst1*_4_Control	0	1.38
*pithless1_pEW398-sst1*_6_Control	0	1.50
*pithless1_pEW398-sst1*__4.1	1	5.00
*pithless1_pEW398-sst1*__4.3	1	5.00
*pithless1_pEW398-sst1*__4.4	1	5.00
*pithless1_pEW398-sst1*__4.5	2 or 4	5.00
*pithless1_pEW398-sst1*__4.6	2	5.00
*pithless1_pEW398-sst1*__6.1	1	5.00
*pithless1_pEW398-sst1*__7.1	4+	5.00
*pithless1_pEW398-sst1*__7.2	1	1.38
*pithless1_pEW398-sst1*__6.2	4+	5.00
*pithless1_pEW398-sst1*__6.3	2	5.00
*pithless1_pEW398-sst1*__6.4	4+	5.00
Kronos_*pEW398-sst1*_2_Control	0	2.75
Kronos_*pEW398-sst1*_2_Control	0	2.75
Kronos_*pEW398-sst1*_3_Control	0	2.50
Kronos_*pEW398-sst1*_2_Control	0	3.13
Kronos_*pEW398-sst1*_9_Control	0	3.38
Kronos_*pEW398-sst1*_2.1	1	5.00
Kronos_*pEW398-sst1*_2.2	3	4.00
Kronos_*pEW398-sst1*_2.3	1	5.00
Kronos_*pEW398-sst1*_2.4	1	5.00
Kronos_*pEW398-sst1*_2.5	1	5.00
Kronos_*pEW398-sst1*_2.6	1	4.88
Kronos_*pEW398-sst1*_2.7	4+	5.00
Kronos_*pEW398-sst1*_2.8	2	5.00
Kronos_*pEW398-sst1*_2.9	3	3.13
Kronos_*pEW398-sst1*_2.11	4+	2.75
Kronos_*pEW398-sst1*_2.12	1	4.75
Kronos_*pEW398-sst1*_2.14	4	5.00
Kronos_*pEW398-sst1*_2.15	2	5.00
Kronos_*pEW398-sst1*_2.16	1	5.00
Kronos_*pEW398-sst1*_2.17	1	5.00
Kronos_*pEW398-sst1*_2.18	3	5.00
Kronos_*pEW398-sst1*_2.19	3	5.00

Kronos_*pEW398-sst1*_2.20	1	5.00
Kronos_*pEW398-sst1*_2.21	4	5.00
Kronos_*pEW398-sst1*_2.23	2	4.13
Kronos_*pEW398-sst1*_2.24	1	5.00
Kronos_*pEW398-sst1*_3.1	1	5.00
Kronos_*pEW398-sst1*_3.3	3	5.00
Kronos_*pEW398-sst1*_3.5	1	4.75
Kronos_*pEW398-sst1*_3.6	4+	5.00
Kronos_*pEW398-sst1*_3.7	1	4.88
Kronos_*pEW398-sst1*_3.8	4	3.00
Kronos_*pEW398-sst1*_3.9	1	5.00
Kronos_*pEW398-sst1*_3.10	1 or 2	5.00
Kronos_*pEW398-sst1*_3.11	1	4.88

Table S4. Genotypic scores for diagnostic KASP markers *usw204* and *usw275* and stem-solidness ratings in a panel of durum and bread wheat cultivars. Average whole stem solidness ratings were assigned for each line (1 to 5 visual rating scale with 1 = very hollow-stemmed, and 5 = very solid-stemmed).

Cultivar	Species	*usw204*	*usw275*	Stem Solidness
pithless1	Durum	1	.	1.2
CDC Fortitude	Durum	0	1	4.6
W9262-260D3	Durum	0	1	4.4
LDN-GB-3B	Durum	0	1	4.6
AC Abbey	Hexaploid Wheat	1	1	1.9
AC Eatonia	Hexaploid Wheat	1	1	3.1
Lillian	Hexaploid Wheat	1	1	2.1
McKenzie	Hexaploid Wheat	1	1	1.5
Glenlea	Hexaploid Wheat	1	1	1.6
CDC Rama	Hexaploid Wheat	1	1	1.7
Unity	Hexaploid Wheat	1	1	1.5
Burnside	Hexaploid Wheat	1	1	1.2
Glencross	Hexaploid Wheat	1	1	1.3
CDN Bison	Hexaploid Wheat	1	1	1.1
CDC Landmark	Hexaploid Wheat	1	1	1.8
Kyle	Durum	1	0	1.3
AC Avonlea	Durum	1	0	1.6
AC Morse	Durum	1	0	1.5
Napoleon	Durum	1	0	1.4
AC Navigator	Durum	1	0	1.9
Strongfield	Durum	1	0	1.6
Commander	Durum	1	0	2.0
CDC Verona	Durum	1	0	1.4
Eurostar	Durum	1	0	1.4
Brigade	Durum	1	0	1.3
Enterprise	Durum	1	0	1.8
Transcend	Durum	1	0	1.6
Langdon	Durum	1	0	1.3
Svevo	Durum	1	0	1.6
Kofa	Durum	1	0	1.4
AC Barrie	Hexaploid Wheat	0	0	1.0
CDC Bounty	Hexaploid Wheat	0	0	1.0
AC Cadillac	Hexaploid Wheat	0	0	1.0
AC Elsa	Hexaploid Wheat	0	0	1.0
Lovitt	Hexaploid Wheat	0	0	1.0
Harvest	Hexaploid Wheat	0	0	1.0
CDC Imagine	Hexaploid Wheat	0	0	1.0
Prodigy	Hexaploid Wheat	0	0	1.0

AC Intrepid	Hexaploid Wheat	0	0	1.0
AC Splendor	Hexaploid Wheat	0	0	1.1
Journey	Hexaploid Wheat	0	0	1.0
AC Superb	Hexaploid Wheat	0	0	1.0
CDC Teal	Hexaploid Wheat	0	0	1.0
CDC Merlin	Hexaploid Wheat	0	0	1.0
5500HR	Hexaploid Wheat	0	0	1.1
CDC Osler	Hexaploid Wheat	0	0	1.0
5600HR	Hexaploid Wheat	0	0	1.0
CDC Go	Hexaploid Wheat	0	0	1.0
5601HR	Hexaploid Wheat	0	0	1.0
CDC Alsask	Hexaploid Wheat	0	0	1.0
Roblin	Hexaploid Wheat	0	0	1.0
Katepwa	Hexaploid Wheat	0	0	1.1
AC Crystal	Hexaploid Wheat	0	0	1.1
Marquis	Hexaploid Wheat	0	0	1.0
AC Foremost	Hexaploid Wheat	0	0	1.0
Red Fife	Hexaploid Wheat	0	0	1.0
AC Taber	Hexaploid Wheat	0	0	1.1
5700PR	Hexaploid Wheat	0	0	1.0
5701PR	Hexaploid Wheat	0	0	1.0
AC Vista	Hexaploid Wheat	0	0	1.4
Snowbird	Hexaploid Wheat	0	0	1.0
AC Andrew	Hexaploid Wheat	0	0	1.0
CDC Walrus	Hexaploid Wheat	0	0	1.1
Infinity	Hexaploid Wheat	0	0	1.0
Waskada	Hexaploid Wheat	0	0	1.0
CDC Abound	Hexaploid Wheat	0	0	1.0
5602HR	Hexaploid Wheat	0	0	1.0
Alvena	Hexaploid Wheat	0	0	1.2
5702PR	Hexaploid Wheat	0	0	1.0
Goodeve	Hexaploid Wheat	0	0	1.0
Snowstar	Hexaploid Wheat	0	0	1.0
Helios	Hexaploid Wheat	0	0	1.0
Bhishaj	Hexaploid Wheat	0	0	1.0
Kane	Hexaploid Wheat	0	0	1.2
Somerset	Hexaploid Wheat	0	0	1.0
5603HR	Hexaploid Wheat	0	0	1.0
Minnedosa	Hexaploid Wheat	0	0	1.0
Shaw	Hexaploid Wheat	0	0	1.0
Stettler	Hexaploid Wheat	0	0	1.0
Glenn	Hexaploid Wheat	0	0	1.0
Carberry	Hexaploid Wheat	0	0	1.0

Muchmore	Hexaploid Wheat	0	0	1.0
5604HR CL	Hexaploid Wheat	0	0	1.0
CDC Stanley	Hexaploid Wheat	0	0	1.0
CDC Kernen	Hexaploid Wheat	0	0	1.0
CDC Utmost	Hexaploid Wheat	0	0	1.0
CDC Thrive	Hexaploid Wheat	0	0	1.0
SY985	Hexaploid Wheat	0	0	1.0
Vesper	Hexaploid Wheat	0	0	1.0
NRG010	Hexaploid Wheat	0	0	1.0
Sadash	Hexaploid Wheat	0	0	1.0
Neepawa	Hexaploid Wheat	0	0	1.0

References

1. Nilsen KT, *et al.* (2017) High density mapping and haplotype analysis of the major stem-solidness locus *SSt1* in durum and common wheat. *PLoS ONE.* 12:e0175285. 10.1371/journal.pone.0175285.
2. Jordan KW, *et al.* (2015) A haplotype map of allohexaploid wheat reveals distinct patterns of selection on homoeologous genomes. *Genome Biol.* 16:48. 10.1186/s13059-015-0606-4.
3. Bolger AM, Lohse M, & Usadel B (2014) Trimmomatic: a flexible trimmer for Illumina sequence data. *Bioinformatics* 30:2114-2120.
4. Scofield GN, *et al.* (2009) Starch storage in the stems of wheat plants: localization and temporal changes. *Ann Bot* 103:859-868.
5. Untergasser A, *et al.* (2012) Primer3--new capabilities and interfaces. *Nucleic Acids Res.* 40:e115-e115. 10.1093/nar/gks596.
6. Voorrips RE (2002) MapChart: software for the graphical presentation of linkage maps and QTLs. *J Hered* 93:77-78.
7. Maccaferri M, *et al.* (2019) Durum wheat genome highlights past domestication signatures and future improvement targets. *Nat Genet* 51:885-895.
8. Appels R, *et al.* (2018) Shifting the limits in wheat research and breeding using a fully annotated reference genome. *Science.* 361:eaar7191. 10.1126/science.aar7191.
9. Pozniak CJ, Nilsen KT, Clarke JM, & Beres BL (2015) CDC Fortitude durum wheat. *Can J Plant Sci* 95:1013-1019.
10. Howells RM, Craze M, Bowden S, & Wallington EJ (2018) Efficient generation of stable, heritable gene edits in wheat using CRISPR/Cas9. *BMC Plant Biol.* 18:215. 10.1186/s12870-018-1433-z.
11. McElroy D, Zhang W, Cao J, & Wu R (1990) Isolation of an efficient actin promoter for use in rice transformation. *Plant Cell* 2:163-171.
12. Risacher T, Craze M, Bowden S, Paul W, & Barsby T (2009) Highly efficient agrobacterium-mediated transformation of wheat via in planta inoculation. *Transgenic Wheat, Barley and Oats: Production and Characterization Protocols*, eds Jones HD & Shewry PR (Humana Press, Totowa, NJ), pp 115-124.
13. Nilsen KT, Clarke JM, Beres BL, & Pozniak CJ (2016) Sowing density and cultivar effects on pith expression in solid-stemmed durum wheat. *Agron J* 108:219-228.
14. Zhang M, *et al.* (2012) Preparation of megabase-sized DNA from a variety of organisms using the nuclei method for advanced genomics research. *Nat Protoc* 7:467-478.
15. Hwang G-H, *et al.* (2018) Web-based design and analysis tools for CRISPR base editing. *BMC Bioinformatics.* 19:542. 10.1186/s12859-018-2585-4.
16. De Coster W, D'Hert S, Schultz DT, Cruts M, & Van Broeckhoven C (2018) NanoPack: visualizing and processing long-read sequencing data. *Bioinformatics* 34:2666-2669.
17. Li H (2016) Minimap and miniasm: fast mapping and de novo assembly for noisy long sequences. *Bioinformatics* 32:2103-2110.
18. Li H (2018) Minimap2: pairwise alignment for nucleotide sequences. *Bioinformatics* 34:3094-3100.
19. Koren S, *et al.* (2017) Canu: scalable and accurate long-read assembly via adaptive k-mer weighting and repeat separation. *Genome Res* 27:722-736.
20. Dobin A, *et al.* (2012) STAR: ultrafast universal RNA-seq aligner. *Bioinformatics* 29:15-21.
21. Pertea M, Kim D, Pertea GM, Leek JT, & Salzberg SL (2016) Transcript-level expression analysis of RNA-seq experiments with HISAT, StringTie and Ballgown. *Nat Protoc.* 11:1650. 10.1038/nprot.2016.095.

22. Langfelder P & Horvath S (2008) WGCNA: an R package for weighted correlation network analysis. *BMC Bioinformatics.* 9:559. 10.1186/1471-2105-9-559.
23. Spurr AR (1969) A low-viscosity epoxy resin embedding medium for electron microscopy. *J Ultrastruct Res* 26:31-43.
24. Bagasra O (2007) Protocols for the in situ PCR-amplification and detection of mRNA and DNA sequences. *Nat Protoc* 2:2782-2795.
25. Athman A*, et al.* (2014) Protocol: a fast and simple in situ PCR method for localising gene expression in plant tissue. *Plant Methods.* 10:29. 10.1186/1746-4811-10-29.
26. Evans AM, DeHaven CD, Barrett T, Mitchell M, & Milgram E (2009) Integrated, Nontargeted Ultrahigh Performance Liquid Chromatography/Electrospray Ionization Tandem Mass Spectrometry Platform for the Identification and Relative Quantification of the Small-Molecule Complement of Biological Systems. *Anal Chem* 81:6656-6667.
27. Oliver MJ*, et al.* (2011) A sister group contrast using untargeted global metabolomic analysis delineates the biochemical regulation underlying desiccation tolerance in *Sporobolus stapfianus*. *Plant Cell* 23:1231-1248.
